# Reductive TCA cycle catalyzed by wild-type IDH2 promotes acute myeloid leukemia and is a metabolic vulnerability for potential targeted therapy

**DOI:** 10.1186/s13045-022-01245-z

**Published:** 2022-03-21

**Authors:** Peiting Zeng, Wenhua Lu, Jingyu Tian, Shuang Qiao, Jiangjiang Li, Christophe Glorieux, Shijun Wen, Hui Zhang, Yiqing Li, Peng Huang

**Affiliations:** 1 State Key Laboratory of Oncology in South China, Collaborative Innovation Center for Cancer Medicine, Sun Yat-sen University Cancer Center, Guangzhou, 510060 China; 2 Metabolic Innovation Center, Sun Yat-sen University, Guangzhou, 510080 China; 3 School of Biomedical and Pharmaceutical Sciences, Guangdong University of Technology, Guangzhou, 510006 China; 4 Department of Hematology, Sun Yat-sen Memorial Hospital, Sun Yat-sen University, Guangzhou, 510120 China

**Keywords:** Acute myeloid leukemia, Wild-type IDH2, TCA cycle, Alpha-ketoglutarate, Lipid synthesis, c-Myc

## Abstract

**Background:**

Isocitrate dehydrogenase-2 (IDH2) is a mitochondrial enzyme that catalyzes the metabolic conversion between isocitrate and alpha-ketoglutarate (α-KG) in the TCA cycle. IDH2 mutation is an oncogenic event in acute myeloid leukemia (AML) due to the generation of 2-hydroxyglutarate. However, the role of wild-type IDH2 in AML remains unknown, despite patients with it suffer worse clinical outcome than those harboring mutant type.

**Methods:**

IDH2 expression in AML cell lines and patient samples was evaluated by RT-qPCR, western blotting and database analyses. The role of wild-type IDH2 in AML cell survival and proliferation was tested using genetic knockdown and pharmacological inhibition in AML cells and animal models. LC–MS, GC–MS, isotope metabolic tracing, and molecular analyses were performed to reveal the underlying mechanisms.

**Results:**

We found that wild-type IDH2 was overexpressed in AML and played a major role in promoting leukemia cell survival and proliferation in vitro and in vivo. Metabolomic analyses revealed an active IDH2-mediated reductive TCA cycle that promoted the conversion of α-KG to isocitrate/citrate to facilitate glutamine utilization for lipid synthesis in AML cells. Suppression of wild-type IDH2 by shRNA resulted in elevated α-KG and decreased isocitrate/citrate, leading to reduced lipid synthesis, a significant decrease in c-Myc downregulated by α-KG, and an inhibition of AML viability and proliferation. Importantly, pharmacological inhibition of IDH2 showed significant therapeutic effect in mice inoculated with AML cells with wt-IDH2 and induced a downregulation of C-MYC in vivo.

**Conclusions:**

Wt-IDH2 is an essential molecule for AML cell survival and proliferation by promoting conversion of α-KG to isocitrate for lipid synthesis and by upregulating c-Myc expression and could be a potential therapeutic target in AML.

**Supplementary Information:**

The online version contains supplementary material available at 10.1186/s13045-022-01245-z.

## Background

Acute myeloid leukemia (AML) is a highly aggressive malignant disease characterized by a rapid proliferation and accumulation of abnormal myeloid cells in the bone marrow and peripheral blood, often accompanied by anemia, fatigue, and increased risk of bleeding and infection [1]. Without proper treatment, AML usually exhibits a highly aggressive disease course leading to bone marrow failure and patient death. Despite a better understanding of AML biology in recent years, it still remains one of the most lethal malignancies with a relatively low cure rate and the 5-year survival rate is less than 30% in adult AML patients [1, 2]. Currently, the cause of AML remains unclear. Multiple factors including genetic alterations, chemical exposure, and radiation have been implicated as risk factors of AML [1]. Among the genetic factors, FLT3-ITD, an internal tandem duplications (ITD) in fms-like tyrosine kinase 3 (FLT3), is observed in approximately 20–30% of AML patients and is associated with aggressive disease progression, worse prognosis, high risk of relapse, and shorter overall survival [3]. Recent studies showed that somatic mutations at isocitrate dehydrogenases (IDHs) could also drive the development of AML due to the production of oncometabolite 2-hydroxyglutarate (2-HG) [4]. Interestingly, somatic mutation of the IDH2 isoform seems more common than that of the IDH1 isoform in AML patients [4].

Isocitrate dehydrogenase 2 (IDH2) is a NADP^+^-dependent mitochondrial enzyme that catalyzes the interconversion between isocitrate and α-ketoglutarate (α-KG) in the tricarboxylic acid (TCA) cycle and thus plays a major role in cellular metabolism [5, 6]. The metabolic flow from isocitrate to α-KG is an oxidative process of the TCA cycle and is usually considered as the normal (forward) metabolic flow direction, whereas the “reverse” flow from α-KG to isocitrate represents a reductive TCA cycle segment. Beside its essential function in cellular metabolism, IDH2 also plays an important role in epigenetic regulation mediated by α-KG, in redox signaling via NADPH/NADP^+^ metabolism, and in affecting the α-KG-dependent DNA repair process [6, 7]. Mutations in IDH2 have been observed in several human cancers, especially in low-grade gliomas and AML patients [8, 9]. Biochemically, the production of 2-HG by the mutated IDH2 enzyme has been considered as a key oncogenic mechanism due to its ability to compete with α-KG to alter the epigenetic landscape of the cells [10, 11]. These new discoveries have led to the development of drugs that specifically target mutant IDH2 for clinical treatment of AML [12, 13].

Interestingly, malignant cells bearing mutant IDH2 were often associated with better responses to treatment compared with those harboring wild-type (wt) IDH2 [14]. Recent studies also found that AML patients with IDH2 mutations were associated with improved overall survival [15, 16] and tend to be correlated with a favorable prognosis compared with those with wt-IDH2 [17–19], suggesting a possibility that the wild-type enzyme could potentially provide certain metabolic advantage to leukemia cells and promote AML progression with poor outcome. The exact role of wt-IDH2 in AML development, however, remains largely unknown. The main goals of the present study were to evaluate the potential effect of wt-IDH2 on AML cell viability and proliferation in vitro and in vivo, and to investigate the underlying mechanisms. Our study has led to significant new findings on the role of wt-IDH2 in promoting the reductive TCA cycle to facilitate the utilization of glutamine for fatty acid synthesis in AML cells, and also gained significant insights into the novel function of wt-IDH2 in regulation of c-Myc expression via α-KG. These new findings together advance the understanding of AML biology with respect to its metabolic characteristics and c-Myc regulation and provide a basis for developing new therapeutic strategy that target wt-IDH2 for AML treatment.

## Methods

### Cell culture

U937, MV-4-11 and MOLM-13 human leukemia cell lines were obtained from DSMZ (German Collection of Microorganisms and Cell Cultures, Braunschweig, Germany). THP-1 cell line was obtained from the American Type Culture Collection (ATCC) (Manassas, VA, USA). HEK-293T, HL-60 and Kasumi-1 were obtained from the Cell Bank of the Chinese Academy of Sciences (Shanghai, China). ML-1 cells were maintained in culture as we described previously [20]. Peripheral blood from patients with primary human AML and normal bone marrow were collected at Sun Yat-Sen Memorial Hospital (Guangzhou, China) after obtaining informed consent. Clinical characteristics of the patients are summarized in Additional file 1: Table S1. Peripheral blood mononuclear cells (PBMCs) and bone marrow cells (BMCs) were isolated by density gradient centrifugation using Ficoll-Paque^™^ Plus (GE Healthcare Bio-Sciences, Uppsala, Sweden) according to the manufacturer’s instructions. AML cell lines and primary human AML cells were cultured in RPMI-1640 (Gibco, Waltham, MA, USA) supplemented with 10% fetal bovine serum (FBS) (Gibco). Bone marrow cells were cultured in Iscove's Modified Dulbecco's Medium (IMDM) (Gibco) supplemented with 15% FBS. HEK-293T cells were cultured in Dulbecco’s modified Eagle’s medium (DMEM) (Gibco) with 10% FBS. All cell lines were cultivated at 37 °C in a humidified incubator with 5% CO_2_ (Thermo Fisher Scientific, Rockford, IL, USA). All cell lines are not among commonly misidentified cell lines and were regularly tested for mycoplasma contamination using LookOut mycoplasma PCR detection kit (Sigma-Aldrich, Saint Louis, MO, USA) to ensure all cells were mycoplasma-free.

### Cell viability assay

Cell viability was measured using MTS assay (3-(4,5-dimethylthiazol-2-yl)-5-(3-carboxymethoxyphenyl)-2-(4-sulfophenyl)-2*H*-tetrazolium, inner salt and PMS (phenazine methosulfate)) as previously reported [21]. In brief, 20,000 cells were seeded in a 96-well plate and treated with the indicated doses of IDH2 inhibitor, AGI-6780 (Selleck, Houston, TX77014, USA) for 72 h and stained for 4 h. The optical density at 490 nm was determined using a Multiskan plate reader (Thermo Fisher Scientific).

### Construction of knockdown (KD) and overexpression (OE) systems

For specific knockdown of IDH2, shRNA-containing plasmids GV-248-sh-IDH2#1, GV-248-sh-IDH2#2, and the control vector GV-248 (Genechem, Shanghai, China) were used. Overexpression plasmid LV105-IDH2 and control vector LV105 were purchased from GeneCopoeia (Rockville, USA). For specific knockdown of c-Myc, shRNA-containing plasmids pLKO.1-sh-MYC and the control vector pLKO.1 (Public Protein/Plasmid Library, Nanjing, China) were used. For the production of lentiviruses, the target plasmids were separately co-transfected with plasmids expressing viral *gag* and *pol* (psPAX2, Addgene, Watertown, MA, USA), and *vsv-g* (pMD2.G, Addgene) genes into HEK-293T cells, using jetPRIME transfection reagents (Polyplus, Illkirch, France) according to the manufacturer’s instructions. The shRNA sequences are listed in Additional file 2: Table S2. The lentiviral particles were harvested at 48 h post-transfection from the supernatant fraction. Cells were infected with lentiviruses in the presence of 8 µg/mL polybrene (Sigma-Aldrich) and then centrifuged at 32 °C, 200 g for 60 min. Cells were resuspended with RPMI-1640 supplemented with 10% FBS and incubated at 37 °C in a humidified incubator with 5% CO_2_. After 72 h, the infected cells were selected with 0.5 mg/mL puromycin (Selleck).

### Small interfering RNA (siRNA) transfection

Three pairs of siRNA primers (si-IDH2) were used to knockdown IDH2, and a non-targeting control siRNA (si-NC) was used as negative control (Guangzhou Ribobio Co., Ltd., Guangzhou, China). The siRNA sequences are listed in Additional file 2: Table S2. U937 and ML-1 cells were transfected with 100 nM si-IDH2 or si-NC using Neon^®^ Transfection System (Invitrogen, Grand Island, NY, USA), according to the manufacturer’s instructions.

### Protein extraction and western blotting

Cells were harvested with lysis buffer (5 g SDS, 0.37 g EDTA, 0.29 g NaCl, and 1 mL 1 M Tris–HCl (pH 7.5) in 100 mL H_2_O) supplemented with protease inhibitor cocktail (Thermo Fisher Scientific). The protein concentration was determined with the bicinchoninic acid (BCA) protein assay kit (Thermo Fisher Scientific). Protein samples were run on a standard SDS-PAGE gel and transferred to PVDF membranes (Millipore, Austin, Texas, USA). Membranes were blocked with 5% non-fat dry milk and incubated overnight at 4 °C with primary antibodies, which are listed in Additional file 2: Table S2. The membranes were then incubated with appropriate HRP-conjugated secondary antibodies (Abcam, Cambridge, MA, USA), and the signals were detected by the ECL detection system (Bio-rad Laboratories, Richmond, CA, USA).

### Cell proliferation and cloning formation assay

For cell proliferation assay, 5 × 10^4^ cells were seeded into 6-well plates and cell numbers were counted every two days using an automated cell counter (Cellometer AutoT4, Nexcelom, USA). For the clonogenic assay, cells were seeded in methylcellulose medium (MethoCult H4230, Stem Cell Technologies) according to the manufacturer’s instructions. 1 × 10^4^ cells in 0.3 mL IMDM (Gibco) with 2% FBS were added into 3 mL of methylcellulose medium, and mixed thoroughly. Three 3.5-cm dishes were plated with 1 mL of the cell suspension each. Colonies were counted after 10 days of incubation under the optical microscope (Olympus CKX31-A12PHP, Japan).

### In vivo xenograft studies

In vivo experiments using mice were reviewed and approved by the Institutional Animals Care and Use Committee of Sun Yat-sen University Cancer Center (Guangzhou, China). At the beginning of the experiment, mice were randomly assigned to each group and were performed in an open-labeled manner. To investigate the role of wild-type IDH2 in AML proliferation, U937 (4 × 10^6^) or ML-1 (2 × 10^6^) cells with or without IDH2 knockdown were injected subcutaneously into the right flanks of female BALB/c nude mice (age 7–8 weeks) (Vital River Laboratory Animal Technology, Beijing, China). Tumor growth was recorded by measuring the length and width of the tumors every two days using a caliper. Tumor size was calculated using the formula (length × width^2^)/2. To evaluate the therapeutic effect of IDH2 inhibitor AGI-6780 in vivo, ML-1 cells (2 × 10^6^) were injected subcutaneously into the right flanks of female BALB/c nude mice (age 7–8 weeks), which were then randomized into two groups to receive the following treatments: (1) Solvent control (PBS containing 10% DMSO and 10% Cremophor EL); (2) AGI-6780 (50 mg/kg in 100 µL solvent, i.p. daily). Treatment was initiated when the tumor volume was about 100 mm^3^. Tumor growth was recorded by measuring the length and width of the tumors every two days using a caliper. Tumor size was calculated using the formula (length × width^2^)/2. The tumors were harvested and weighed at the experimental endpoint.

### Flow cytometry analysis of apoptosis

For analysis of apoptosis, C-MYC knockdown cell death, and the number of apoptotic cells after AGI-6780 and dimethyl-αKG treatment, was analyzed by FITC-Annexin V/PI assay kit (BD, Franklin Lakes, NJ, USA). IDH2 knockdown cell death was analyzed using APC-Annexin V/7AAD assay kit (BD) according to the manufacturer’s instructions. Stained cells were analyzed with Beckman flow cytometer (Beckman Coulter, Miami, FL, USA).

In order to quantify intracellular ROS levels, cells were stained with 5 μM DCFH-DA for 30 min at 37 °C in dark and analyzed by CytoFLEX flow cytometer (Beckman, USA).

### RNA extraction and quantitative real-time PCR

Total RNA was isolated using TRIzol reagents according to the manufacturer’s instructions (Thermo Fisher Scientific). Total RNA was reverse-transcribed into cDNA using Reverse Transcription System Kit (Takara BIO INC, Kusatsu, Shiga, Japan). cDNA was used for quantitative Real-Time PCR (Bio-Rad CFX96 real-time PCR detection system, Bio-Rad Laboratories) using specific primers and SYBR Premix Ex Taq RNAse H^+^ kit (Takara) to determine target mRNA levels. The primers sequences used in this study were listed in Additional file 2: Table S2 and produced by Sangon Biotech (Shanghai, China). The 2^−ΔΔCt^ method was used to normalize mRNA levels to β-actin (reference gene).

### Measurement of cellular oxygen consumption and acid production

XFe96 Extracellular Flux Analyzer (Seahorse Bioscience, North Billerica, MA, USA) was used to quantify real-time oxygen consumption rate (OCR) and extra cellular acidification rate (ECAR) according to the manufacturer's user guides. The Seahorse cartridge was placed in the XF calibrant and incubated overnight in a CO2-free incubator at 37 °C. Cells were seeded in a Cell-Tak (Corning) pretreated Seahorse 96-well culture microplate at density 1.0 × 10^5^ per well. For OCR detection, cells were cultured in the base medium with 1 mM pyruvate, 2 mM glutamine, and 10 mM glucose on the day of the assay and incubated for 1 h in a CO_2_-free incubator at 37 °C. Injections of oligomycin (1 µM, ATP synthase inhibitor), FCCP (0.5 µM for U937 cell line and 1 µM for ML-1 cell line, mitochondrial uncoupler), and rotenone/antimycin A (0.5 µM, complex I/III inhibitors) were performed sequentially as indicated. For ECAR detection, the medium was changed to base medium supplemented with 2 mM glutamine on the day of the assay and incubated for 1 h in a CO_2_-free incubator at 37 °C. Injections of glucose (10 mM), oligomycin (1 µM), and 2-DG (50 mM) were performed sequentially as indicated.

### Cellular glucose uptake and lactate production

Cells (3 × 10^5^) were seeded in 24-well plates and cultured for 24 h. Supernatants from each well were centrifuged and collected for analyzing glucose and lactate contents using a Yellow Springs Instrument 2950D-1 (YSI Inc./Xylem Inc., Yellow Springs, USA). Results were normalized to cell numbers at the endpoint.

### Measurement of cellular ATP

Cellular ATP concentration was detected using an ATP-based CellTiter-Glo Luminescent Cell Viability Kit (Promega, Madison, WI, USA). Cells (2 × 10^4^) were seeded in 96-well plates in an incubator for 4 h. CellTiter-Glo (100 µL) reagents were added to each well and rocked for 2 min to lyse cells for ATP detection. The samples were kept at room temperature for another 10 min. The ATP contents were recorded as luminescent signal, using a luminescent plate reader (Thermo Fisher Scientific).

### Immunohistochemistry (IHC)

The procedures for IHC were performed as described previously [22]. The primary antibodies used in this study are listed in Additional file 2: Table S2. DAB (3,3′-diaminobenzidine) was then applied as a substrate to reveal the antigen, and hematoxylin was used for counterstaining.

### Terminal deoxynucleotidyl transferase dUTP nick end labeling (TUNEL) assay

AML tissue slices were stained by a TUNEL assay kit according to the manufacturer’s instructions (KeyGEN BioTECH. Nanjing, China) to determine cell death in vivo. TUNEL-positive cells were imagined using a Nikon Eclipse 800 microscope. Three random slices from each group were quantified.

### Metabolic flux analysis

Metabolic flux using uniformly ^13^C-labeled ([U-^13^C_5_]) glutamine was performed as previously published [23, 24]. Cells were cultured (3 × 10^6^ per 60 mm-dish) in glutamine-free RPMI-1640 supplemented with 10% FBS and 2 mM ^13^C-labeled glutamine (Cambridge Isotope Laboratories, Inc. Andover, MA, USA). After 24 h, cells were rinsed twice with ice-cold 0.9% (w/v) NaCl solution and then collected into new tubes. 500 µL of − 80 °C methanol was added to quench cellular metabolism, 200 µL of ice-cold water containing 1 µg norvaline internal standard, and 500 µL of − 20 °C chloroform containing 1 µg palmitate were added to each tube. After vortexing and centrifugation, the top aqueous layer and the bottom organic layer were collected and dried at 4 °C under vacuum drying and nitrogen airflow, respectively. The dry pellets were then stored at − 80 °C until analysis by GC–MS. For derivatization of polar metabolites, dried metabolites were dissolved in 20 µL of 2% (*w*/*v*) methoxyamine hydrochloride in pyridine and held at 37 °C for 60 min. Subsequent conversion to their tert-butyldimethylsilyl (tBDMS) derivatives was accomplished by adding 30 µL MtBSTFA + 1% tBDMCS (Regis Technologies, Inc. Austin Ave Morton Grove, IL, USA) and incubating at 45 °C for 30 min. Fatty acid methyl esters (FAMEs) were generated by dissolving dried fatty acids in 500 µL 2% (v/v) methanolic sulfuric acid and incubating at 50 °C for 2 h. FAMEs were subsequently extracted in 1 mL hexane with 0.1 mL saturated sodium chloride solution. The upper layer was transferred into a new tube and dried under nitrogen airflow. The dried samples were dissolved with 100 µL hexane. The samples were subsequently analyzed by GC/MS (ThermoFisher Scientific Trace 1300 TSQ).

### Quantitative analysis of target metabolites

In order to quantify intracellular metabolites (α-KG, isocitrate, citrate, etc.), cells (1 × 10^7^) were collected and washed twice with cold 0.9% (*w*/*v*) NaCl solution. 800 µL of cold methanol was added to quench cellular metabolism, followed by addition of 200 µL of ice-cold water. After vortexing and centrifugation, the upper layer fraction was analyzed by UHPLC-MS/MS system (Agilent 1290 Infinity II, Santa Clara, California, USA).

### Statistical analysis

All experiments were performed at least three times (3 separate repeats). The student’s *t*-test was used to evaluate the statistical significance of the difference between two groups of samples. When there were more than two independent groups, ANOVA was used to compare the means of samples. Dunnett post hoc tests were performed when ANOVA was significant. General linear model with repeated measures analysis of variance was used for in vivo experiments. Statistical analyses were performed with GraphPad Prism 8.0 (San Diego, CA, USA) and SPSS 20.0 (Chicago, IL, USA). All tests were two-tailed, and a *p* value of 0.05 or less was considered statistically significant.

## Results

### Wild-type IDH2 is overexpressed in AML and promotes leukemia cell proliferation

**Fig. 1:**
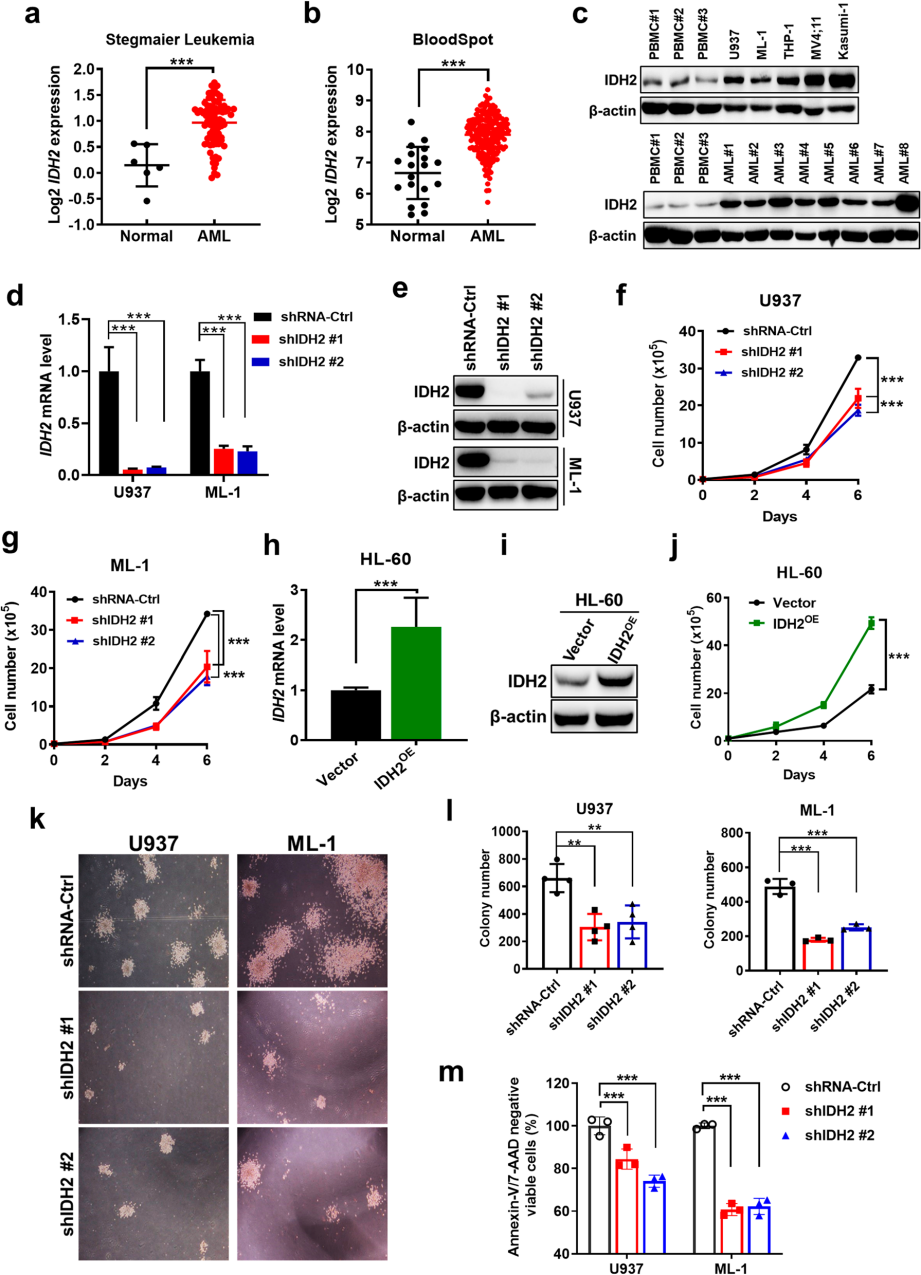
Over-expression of wild-type IDH2 in AML and its effect on leukemia cell proliferation. **a** Comparison of IDH2 mRNA levels in AML (*n* = 80) and normal cells (monocytes and neutrophils, *n* = 6), using the leukemia dataset (Stegmaier Leukemia datasets) available in the Oncomine database. **b** IDH2 mRNA levels in primary AML samples (*n* = 204) in comparison with normal cells (hematopoietic stem cells, *n* = 6; metamyelocytes, *n* = 3; band cells, *n* = 3; polymorphonuclear cells, *n* = 3; monocytes, *n* = 4) available in the BloodSpot datasets. **c** Western blotting of IDH2 in normal human peripheral blood mononuclear cells (PBMC #1–#3), AML cell lines and PBMCs from AML patients with wt-IDH2 (AML#1–#8), β-actin was used as a loading control. **d** Relative mRNA level of IDH2 in U937 and ML-1 cells transfected with control shRNA (shRNA-Ctrl) or IDH2 shRNA (shIDH2#1, shIDH2#2) was measured by RT-qPCR. **e** Western blotting analysis of IDH2 protein in U937 and ML-1 cells transfected with shRNA-Ctrl or IDH2 shRNA. **f**, **g** Measurement of cell proliferation in U937 and ML-1 cells expressing shRNA-Ctrl or IDH2 shRNA. **h** Relative IDH2 mRNA level in HL-60 cells transfected with control vector (Vector) or IDH2 over-expression vector (IDH2^OE^). Expression of mRNA was measured by RT-qPCR. **i** Western blotting analysis of IDH2 protein levels in HL-60 cells transfected with control vector or IDH2 over-expression vector. **j** Comparison of cell proliferation in HL-60 cells transfected with control vector or IDH2 over-expression vector. **k**, **l** Colony formation assay in U937 and ML-1 cells expressing shRNA-Ctrl or IDH2 shRNA. Colonies were counted under inverted microscope. **m** Comparison of percentage of Annexin V/ PI negative cells in U937 and ML-1 transfected with shRNA-Ctrl or IDH2 shRNA for 48 h. The original flow cytometry analysis data are presented as Additional file 3: Fig. S1h. *n* = 3, *mean* ± *SD*; *p < 0.05, **p < 0.01, ***p < 0.001

To evaluate the potential role of wild-type (wt) IDH2 in AML disease development, we first analyzed the expression of IDH2 in primary AML samples in comparison with normal cells, using the leukemia datasets from Oncomine [25] and BloodSpot [26], which contained RNA sequencing (RNA-seq) data from clinical specimens of AML patients. We found that IDH2 expression was significantly higher in AML cells compared to the normal samples (Fig. 1a, b), and patients with higher IDH2 expression exhibited a tendency of poor clinical outcome (Additional file 3: Fig. S1a). Since only a small proportion (approximately 10%) of AML patients have IDH2 mutation [27, 28], the high expression of IDH2 observed in these patient samples seemed to suggest that the elevated expression was mainly from wt-IDH2. We then further examined the expression of IDH2 in AML cell lines and primary AML cells that had been confirmed to harbor wt-IDH2 in comparison with normal peripheral blood mononuclear cells (PBMCs), and consistently found that wt-IDH2 protein expression was substantially higher in AML cells compared with normal PBMCs (Fig. 1c; Additional file 3: Fig. S1b, c). The high expression of wt-IDH2 in AML cell lines and primary patient samples suggests that this molecule might be important for AML cells.

Multiple experimental approaches were then used to directly test the role of wt-IDH2 in AML cells survival and proliferation. First, shRNA was used to specifically knock down IDH2 expression in two myeloid leukemia lines (U937 and ML-1) that harbor wild-type IDH2 gene (Additional file 3: Fig. S1d) and then examined the impact of decreased IDH2 expression on AML cells survival and proliferation. RT-qPCR and western blotting analyses showed that IDH2 shRNA effectively reduced the mRNA and protein levels of IDH2 by 80–90% (Fig. 1d, e) and did not significantly affect the expression of IDH1 and IDH3 (Additional file 3: Fig. S1e–g), confirming the efficiency and specificity of the IDH2-shRNA knockdown (KD). The suppression of IDH2 expression significantly slowed down AML cell proliferation (Fig. 1f, g). We then performed a gain-of-function experiment by overexpressing wt-IDH2 in HL-60 cells, which exhibited relatively low background IDH2 expression. We found that ectopic expression of wt-IDH2 accelerated HL-60 growth (Fig. 1h–j). Colony formation assay showed that a knockdown of wt-IDH2 impaired the AML capacity to form colonies (Fig. 1k, l). Flow cytometry analysis revealed that a transient knockdown of IDH2 for 48 h increased the proportion of apoptosis-positive cells (Fig. 1m; Additional file 3: Fig. S1h). Consistent with these findings, transcriptomic analyses using RNA-Seq showed an enrichment of apoptotic pathway in U937 cells with IDH2 knockdown (Additional file 3: Fig. S1i). Together, these data suggest that wt-IDH2 might play an important role in AML cell survival and proliferation.

**Fig. 2:**
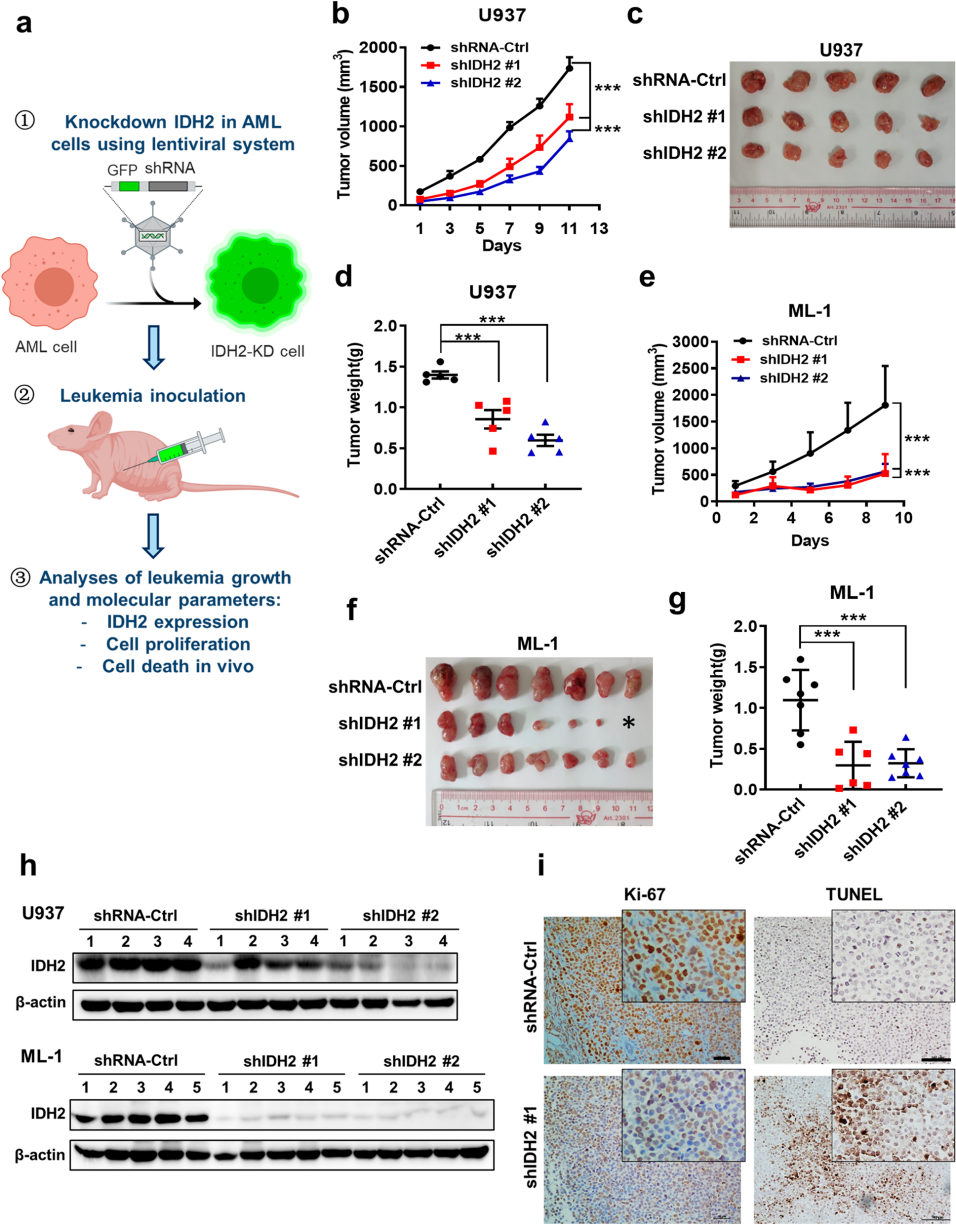
Impact of IDH2 knockdown on AML growth in vivo. **a** Outline of in vivo study design using AML cell models (U937 and ML-1) with shRNA-Ctrl and shIDH2#1 or shIDH2#2. **b**–**d** Tumor growth in athymic nude mice inoculated with U937 cells harboring shRNA-Ctrl, shIDH2#1 or shIDH2#2 (*n* = 5 per group, *mean* ± *SEM*). Tumor sizes were measured every two days. At the end of the experiment, tumors were isolated, photographed and weighted. **e**–**g** Tumor growth in athymic nude mice inoculated with ML-1 cells harboring shRNA-Ctrl, shIDH2#1 or shIDH2#2 (*n* = 7 per group, *mean* ± *SD*). Tumor sizes were measured every two days. Tumors were photographed and weighted at the end of the experiment. The star * in **f** indicates no tumor formation in this mouse. **h** Representative Western blotting of IDH2 protein expression in tumor tissues isolated from mice inoculated with AML cells harboring shRNA-Ctrl or IDH2 shRNA as indicated. **i** Left panel: representative images of Ki-67 IHC staining of tumor tissues from U937 xenografts with shRNA-Ctrl or IDH2 shRNA as indicated. The scale bars represent 50 μm; Right panel: apoptotic cells in tumor tissues of U937 xenografts with shRNA-Ctrl or IDH2 shRNA detected by terminal deoxynucleotidyl transferase dUTP nick end labeling (TUNEL) assay. The scale bars represent 100 μm. ***p < 0.001

We then used leukemia xenograft models to further evaluate the role of wt-IDH2 in AML proliferation in vivo. As shown in Fig. 2a, U937 or ML-1 cells were first infected with lentivirus carrying IDH2-shRNA or control RNA vectors and then inoculated into athymic mice to test tumor formation and growth. The results showed that IDH2 knockdown significantly reduced tumor growth in both U937 (Fig. 2b–d) and ML-1 (Fig. 2e–g) leukemia models. Western blotting analysis of the tumor tissues confirmed a substantial decrease of IDH2 protein expression in the tumors from mice inoculated with cells harboring IDH2-shRNA (Fig. 2h; Additional file 3: Fig. S2a, b). Interestingly, the level of IDH2 knockdown seemed to be correlated with the degree of tumor growth retardation. Immunohistochemical (IHC) analysis of the tissue sections from the resected tumors showed a decrease in expression of Ki-67, indicating a decrease in cell proliferation in tumors from IDH2-KD AML cells (Fig. 2i, left panels). Terminal deoxynucleotidyl transferase dUTP nick end labeling (TUNEL) assay revealed an increase of apoptosis in tumors with suppressed IDH2 (Fig. 2i, right panels). These data together indicate that IDH2 plays an important role in promoting cancer cell survival and proliferation in vivo.

### IDH2 promotes the reductive TCA cycle to enhance glutamine utilization for fatty acid synthesis

To investigate the possible mechanisms by which wt-IDH2 promotes AML cell proliferation, we first tested the impact of IDH2 on the leukemia cell metabolism based on the enzymatic function of IDH2 in TCA cycle and mitochondrial metabolism. A Seahorse XFe96 extracellular flux analyzer was first used to measure the impact of IDH2 on oxygen consumption rate (OCR) as an indicator of mitochondrial oxidative phosphorylation (OXPHOS) activity, and on extra cellular acidification rate (ECAR) as an indicator of glycolysis. Surprisingly, a knockdown of IDH2 did not significantly alter OCR (Additional file 3: Fig. S3a–c) or ECAR (Additional file 3: Fig. S3d–f), nor did it affect glucose uptake and lactate production (Additional file 3: Fig. S3g–j). Consistent with this unexpected observation, there were no significant changes in cellular ATP level when the wt-IDH2 was knocked down in AML cells by shRNA (Additional file 3: Fig. S3k–l). We also used flow cytometry with DCFDH-DA probe to measure cellular ROS and did not observe a difference in the intracellular ROS after knocking down IDH2 (Additional file 3: Fig. S3m).

**Fig. 3:**
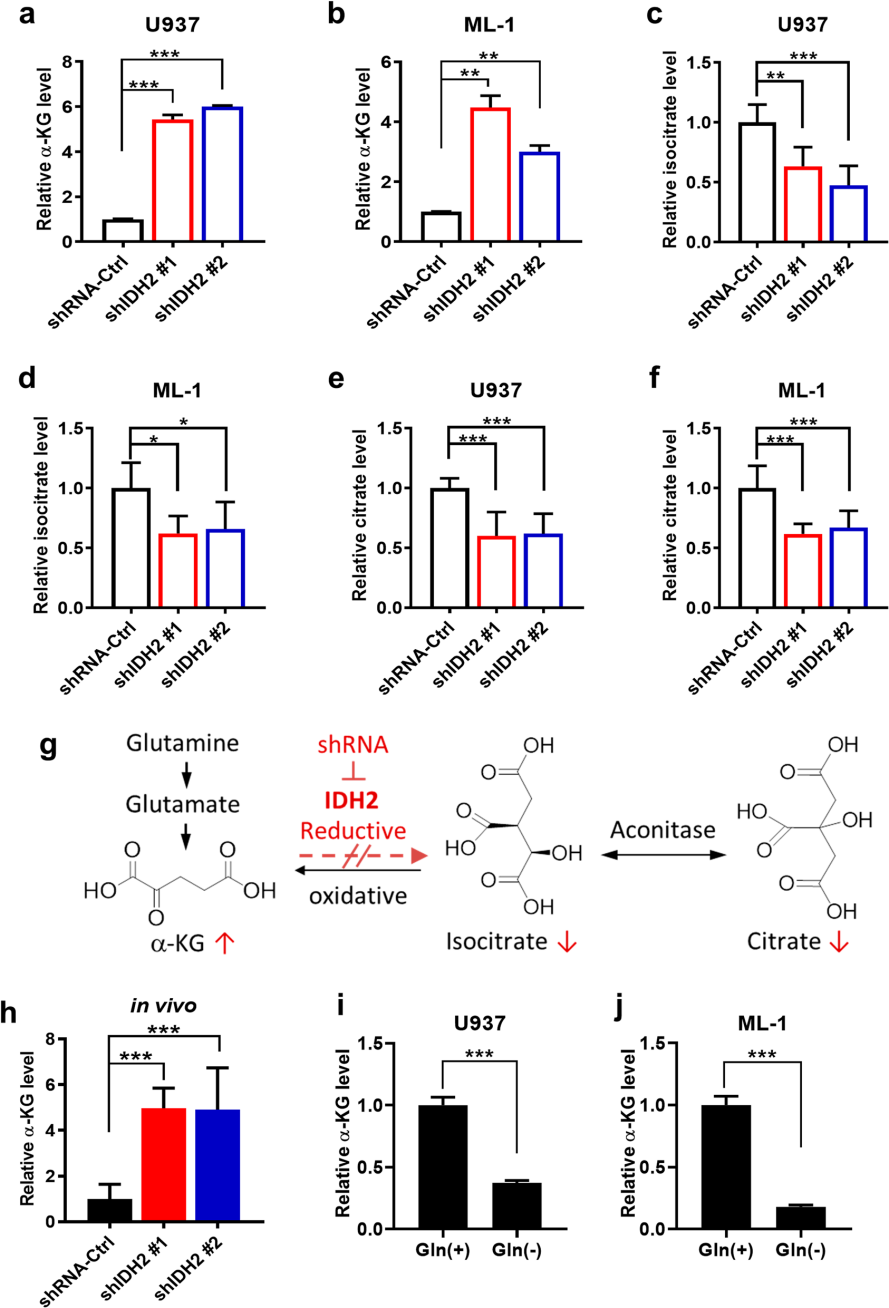
Suppression of IDH2 blocked the conversion of α-KG to isocitrate in reductive TCA cycle. **a**, **b** Quantitation of α-KG in U937 or ML-1 cells harboring shRNA-Ctrl, shIDH2#1 or shIDH2#2. **c**, **d** Levels of isocitrate in U937 or ML-1 cells harboring shRNA-Ctrl, shIDH2#1 or shIDH2#2. **e**, **f** Levels of citrate in U937 or ML-1 cells harboring shRNA-Ctrl, shIDH2#1 or shIDH2#2. **g** Schematic illustration of changes in α-KG, isocitrate, and citrate in AML cells after suppression of active reductive TCA flow using IDH2 shRNA. **h** Quantitation of α-KG in tumor tissues from mice bearing U937 xenografts harboring shRNA-Ctrl, shIDH2#1 or shIDH2#2. **i**, **j** Comparison of α-KG levels in U937 and ML-1 cells cultured with or without glutamine for 24 h. Gln (+): glutamine 2 mM, Gln (−): glutamine free. *n* = 3, *mean* ± *SD*, *p < 0.05, **p < 0.01, ***p < 0.001

The lack of significant effect of IDH2 knockdown on glucose energy metabolism prompted us to explore other potential mechanisms responsible for its impact on AML cell proliferation. Since the main function of IDH2 is to catalyze the metabolic conversion between isocitrate and α-KG [29], we thus used mass spectrometry to quantitatively measure the immediate metabolites of IDH2, including α-KG, isocitrate, and citrate in AML cells with or without IDH2 knockdown. As shown in Fig. 3a, b, IDH2 knockdown by shRNA surprisingly led to a significant increase of α-KG in both U937 and ML-1 cells. This was unexpected since a blockage of isocitrate to α-KG flow of the TCA cycle in forward direction would predict a decrease of α-KG. We then measured cellular isocitrate and citrate levels and found that these two metabolites were significantly decreased in both U937 and ML-1 cells with IDH2 knockdown (Fig. 3c–f). These data together seemed to suggest an active reductive (reverse) TCA cycle in AML cells was highly active to convert α-KG to isocitrate/citrate, and a suppression of IDH2 by shRNA would lead to an accumulation of α-KG and a decrease of isocitrate and citrate, as illustrated in Fig. 3g. Indeed, analysis of tumor tissues also revealed a major increase of α-KG by fivefold in the tumor xenografts with IDH2 knockdown (Fig. 3h), indicating that this occurred both in vitro and in vivo. Based on these observations, we speculated that glutamine might be a key source of α-KG production in AML cells as illustrated in Fig. 3g. To test this possibility, U937 and ML-1 cells were incubated with or without glutamine for 24 h, and cellular α-KG level was measured. The results showed that removal of glutamine from the culture medium led to a profound depletion of cellular α-KG in both cell lines (Fig. 3i, j).

**Fig. 4:**
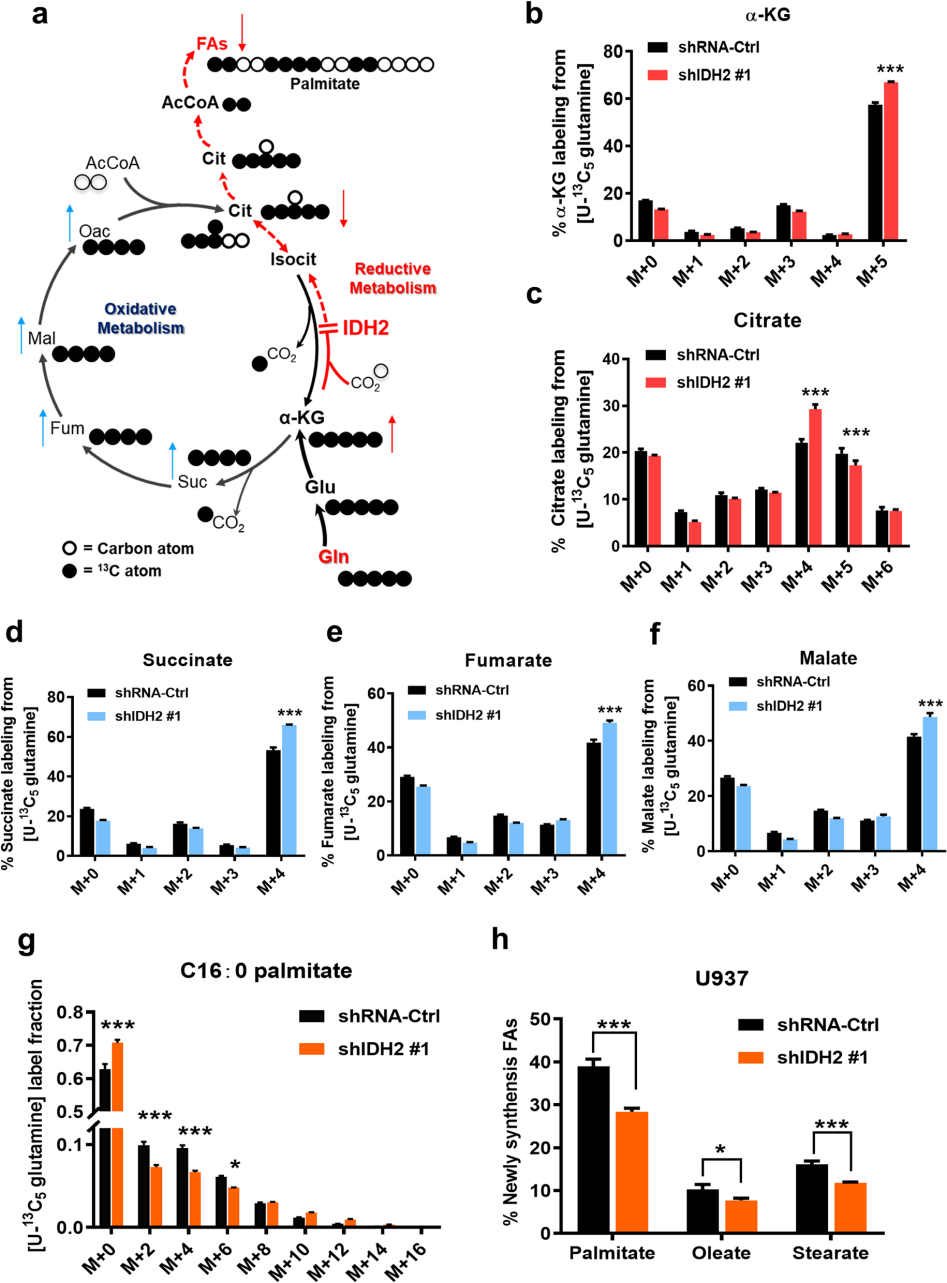
Glutamine metabolic flux analysis in AML cells with or without IDH2 knockdown. **a** Schematic of [U-^13^C_5_] glutamine metabolism tracing experiment. Open circles depict ^12^C atoms and filled circles depict ^13^C atoms. Metabolite abbreviations: Gln: glutamine, Glu: glutamate, α-KG: alpha-ketoglutarate, Isocit: isocitrate, Cit: citrate, AcCoA: acetyl-coenzyme A, FAs: fatty acid synthesis, Oac: oxaloacetate, Mal: malate, Fum: fumarate, Suc: succinate, Pyr: pyruvate, Asp: aspartate. The red arrows indicate the direction of changes in metabolites due to suppression of reductive TCA flow, while the blue arrows indicate the direction of changes in metabolites of the oxidative TCA segment. **b**–**f** Mass isotopomer distribution of the TCA cycle metabolites in U937 cells harboring shRNA-Ctrl or IDH2 shRNA cultured in medium containing [U-^13^C_5_] glutamine for 24 h. **g** Mass isotopomer distribution of palmitate in U937 cells with shRNA-Ctrl or IDH2 shRNA after cultured in medium containing [U-^13^C_5_] glutamine for 24 h. **h** Percentage of newly synthesized palmitate, oleate and stearate in U937 cells with shRNA-Ctrl or IDH2 shRNA after cultured in medium containing [U-^13^C_5_] glutamine for 24 h. *n* = 3, *mean* ± *SD*, *p < 0.05, ***p < 0.001

We then performed metabolic flux analysis, using [U-^13^C_5_] glutamine to evaluate its flux in AML cells with or without IDH2 knockdown (Fig. 4a). The results revealed that a knockdown of IDH2 by shRNA in AML cells caused an increase in M + 5 labeling of α-KG (Fig. 4b), consistent with the observation in Fig. 3a, b. Importantly, there was a significant decrease in the flux from α-KG to citrate through reductive (reverse) TCA cycle, as evidenced by a decrease in M + 5 labeled citrate (Fig. 4c). Interestingly, the flux from α-KG to M + 4 labeling of succinate and its downstream metabolites fumarate and malate increased in the IDH2 knockdown cells (Fig. 4d–f), indicating that the accumulation of α-KG in IDH2-KD cells could force its metabolic α-KG flow in the oxidative direction, and subsequently contribute to an increase of M + 4 labeling of citrate (Fig. 4c). Analysis of ^13^C-labeled palmitate showed a significant decrease in M + 2, M + 4 and M + 6 signals (Fig. 4g), indicating that the overall glutamine flux into fatty acids was suppressed in IDH2 knockdown AML cells. Indeed, we observed a significant decrease in the newly synthesized palmitate, oleate, and stearate after IDH2 knockdown (Fig. 4h). These data together suggest that wt-IDH2 might play an important role in promoting reductive TCA cycle, and thus facilitating de novo fatty acid biosynthesis from glutamine in AML cells.

### α-KG is a key metabolite mediating the effect of IDH2 on cell proliferation and survival

**Fig. 5:**
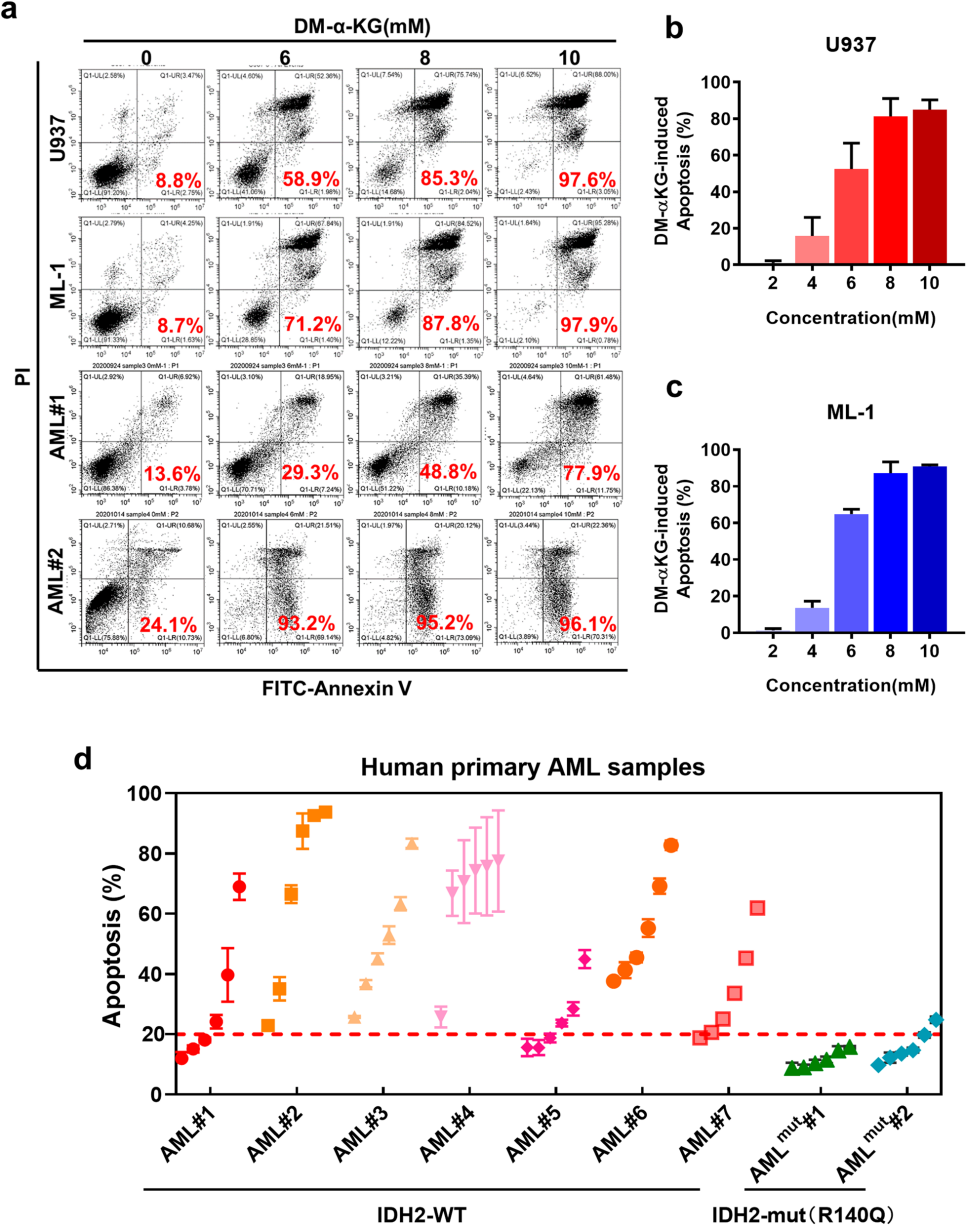
Cytotoxic effect of α-KG in AML cells with wild-type IDH2. **a** Induction of apoptosis by cell-permeable DM-αKG in AML cell lines (U937 and ML-1) and primary AML cells isolated from patients with wt-IDH2 (AML#1 and AML#2). Cells were treated with the indicated concentrations of DM-αKG for 48 h, and apoptosis was measured by flow cytometry analysis of annexin-V positivity. The number inside each panel shows the percentage of dead cells. **b**, **c** Quantitation of the concentration-dependent apoptosis induced by DM-αKG in U937 and ML-1 cells (*n* = 3, *mean* ± *SD*). **d** Apoptosis of human primary AML cells harboring wild-type (*n* = 7) or mutant IDH2 (IDH2-R140Q, *n* = 2) treated with various concentrations (2, 4, 6, 8 and 10 mM) of DM-αKG for 48 h. Apoptosis was measured by flow cytometry analysis of annexin-V positivity

Based on the observations that knockdown IDH2 significantly increased α-KG level in AML cells and suppressed cell proliferation and survival, we next examined the contribution of elevated α-KG to the biological phenotype induced by IDH2 ablation. U937 and ML-1 cells were exposed to various concentrations of cell-permeable α-KG and its impact on the leukemia cells was evaluated. As shown in Fig. 5a–c, treatment of AML cells with 2–8 mM dimethyl-αKG (DM-αKG, a cell-permeable derivative of α-KG) strongly induced apoptosis, suggesting that elevated intracellular α-KG per se was sufficient to have a major impact on AML cell survival, and the cytotoxic effect of IDH2 knockdown could be attributed, at least in part, to the abnormal accumulation of α-KG. To investigate the relevance of these findings in AML, we isolated primary leukemia cells from the peripheral blood of AML patients carrying wt-IDH2 or mutant IDH2 (Additional file 1: Table S1) and treated the leukemia cells with DM-αKG. Consistently, DM-αKG induced significant apoptosis in primary AML cells with wt-IDH2 (Fig. 5a, d; Additional file 3: Fig. S4a, b). However, IDH2 mutant (R140Q) AML cells surprisingly exhibited resistance to DM-αKG treatment (Fig. 5d; Additional file 3: Fig. S4c, d), indicating a major difference between the leukemia cells with wt-IDH2 and those with mutated IDH2.

### Novel role of IDH2 and α-KG in regulation of c-Myc expression

**Fig. 6:**
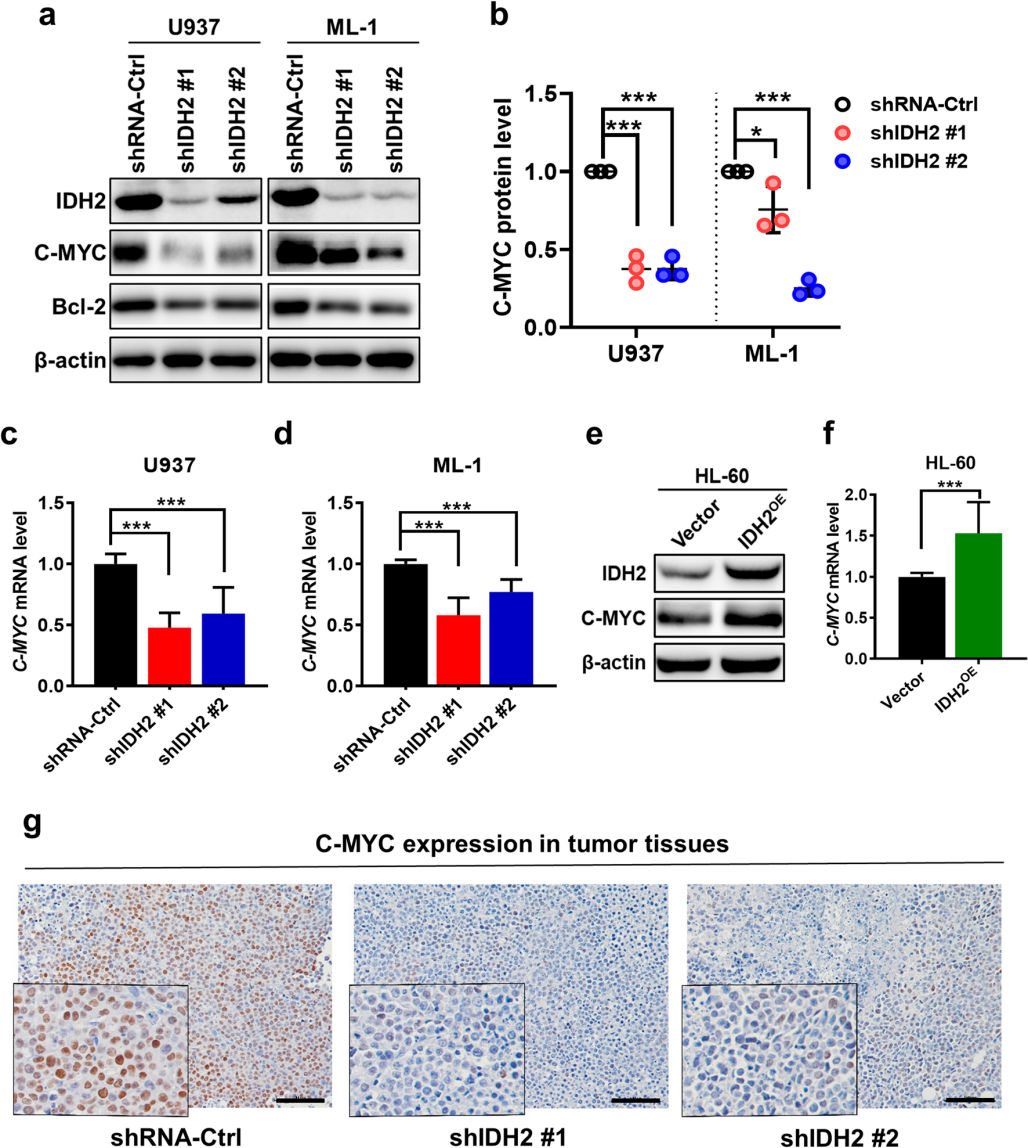
Regulation of c-Myc expression by wild-type IDH2 in AML cells. **a** Effect of IDH2 knockdown on the expression of IDH2, C-MYC and its target gene Bcl-2 in U937 and ML-1 cells. Protein expression was analyzed by Western blotting. **b** Relative protein levels of C-MYC in U937 and ML-1 cells transfected with shRNA-Ctrl or shIDH2 vectors (#1 and #2). Data are representative of three separate experiments. **c** Relative mRNA level of *C-MYC* in U937 cells harboring shRNA-Ctrl or shIDH2 vectors. RNA expression was measured by RT-qPCR. **d** Relative mRNA level of *C-MYC* in ML-1 cells harboring shRNA-Ctrl or shIDH2 vectors. RNA expression was measured by RT-qPCR. **e** Expression of IDH2 protein and C-MYC protein in HL-60 cells transfected with control vector (Vector) or wt-IDH2 expression vector (IDH2^OE^), protein expression was detected by Western blotting. **f** Relative mRNA levels of *C-MYC* in HL-60 cells harboring control vector or IDH2^OE^ vector, measured by RT-qPCR. **g** Representative images of C-MYC immunohistochemistry (IHC) staining of tumor tissues from U937 xenografts harboring shRNA-Ctrl or shIDH2 vectors (#1 or #2). The scale bars represent 100 μm. *n* = 3, *mean* ± *SD*, *p < 0.05, ***p < 0.001

Since *C-MYC* is an important oncogene that plays a major role in leukemia cell survival and cancer metabolism [30, 31], we thus used genetic and biochemical approaches to evaluate the potential link between IDH2 and c-Myc. As shown in Fig. 6a, b, a knockdown of wt-IDH2 by shRNA caused a substantial decrease in C-MYC protein, which was observed in both U937 and ML-1 cell lines. RT-qPCR analysis revealed a significant decrease in *C-MYC* mRNA in AML cells with IDH2 knockdown (Fig. 6c, d), suggesting that the change involved a downregulation at the transcriptional level. Conversely, a forced overexpression of wt-IDH2 in HL-60 cells led to a significant increase in c-Myc mRNA and protein levels (Fig. 6e, f). IHC staining of tumor tissues isolated from mice inoculated with AML cells with or without IDH2 knockdown revealed a major decrease of C-MYC expression in those with IDH2 knockdown (Fig. 6g), indicating that C-MYC down regulation by IDH2 knockdown occurred in vivo.

Interestingly, analysis of the GEPIA database [32] revealed a significant positive correlation between IDH2 and *C-MYC* mRNA expression in 173 AML patient samples (Additional file 3: Fig. S5a), suggesting that the regulation of c-Myc by IDH2 seemed clinically relevant. This was further confirmed by two other datasets with larger AML patients sample sizes carrying wt-IDH2 from cBioPortal database [33, 34] (Additional file 3: Fig. S5b, c). The expression of Bcl-2, a survival molecule known to be regulated by C-MYC [35], was also reduced in the IDH2-KD cells (Fig. 6a). RT-qPCR analysis showed that the expression of multiple c-Myc target genes (*Bcl-2*, *MDM2*, *Cdc25a*, *ELK-1*, *SLC7A11*, etc.) was reduced to various degrees in two AML cell lines with IDH2 knockdown (Additional file 3: Fig. S5d), while increased in HL-60 cells with overexpressing IDH2, further supporting the conclusion that IDH2 regulates c-Myc expression and affect its functions. Considering that C-MYC is a master regulator of cell metabolism and proliferation, we knocked-down c-Myc expression in U937 and ML-1 cell lines, and observed whether the expression of IDH2 was regulated by C-MYC. As shown in Additional file 3: Fig. S6a, knockdown of c-Myc did not change the expression of IDH2, indicating that IDH2 was not directly regulated by C-MYC. Furthermore, knockdown of c-Myc decreased AML cells 72 h-proliferation rate significantly and induced cell apoptosis (Additional file 3: Fig. S6b–d), suggesting the crucial role of c-Myc in the proliferation and survival of AML cells with wt-IDH2.

**Fig. 7:**
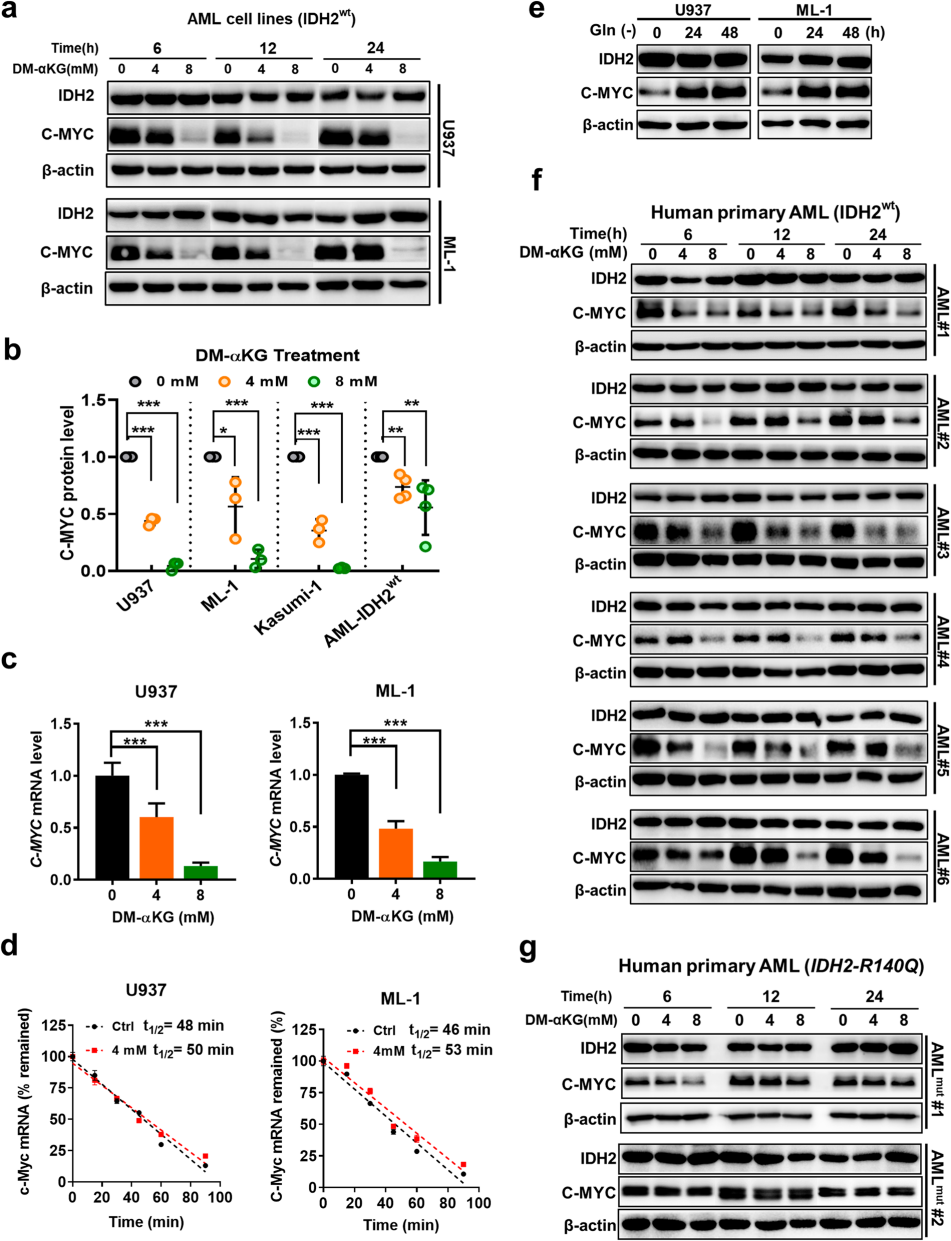
Effect of α-KG on c-Myc expression in AML cell lines and primary leukemia cells. **a** Western blotting analysis of IDH2 and C-MYC protein expression in U937 and ML-1 cells treated with the indicated concentrations of DM-αKG for 6, 12 and 24 h. **b** Relative protein levels of C-MYC in AML cell lines (data from three separate experiments) and primary leukemia cells from AML patients with wild-type IDH2 (*n* = 4) treated in vitro with the indicated concentrations of DM-αKG for 12 h. *p < 0.05, **p < 0.01, ***p < 0.001. **c** Relative mRNA level of *C-MYC* in U937 and ML-1 cells treated with the indicated concentrations of DM-αKG for 6 h. RNA expression was measured by RT-qPCR. ***p < 0.001. **d** Effect of DM-αKG (4 mM) on c-Myc mRNA stability. Cells were pre-treated with DM-αKG for 4.5 h followed by incubation with 5 μg/mL actinomycin D for the indicated time periods (0, 15, 30, 45, 60 and 90 min). The mRNA degradation rate was estimated according to a published method [52]. **e** C-MYC protein expression in U937 and ML-1 cells cultured with or without glutamine (Gln) for 24–48 h as indicated. **f** Western blotting of IDH2 and C-MYC protein expression in primary leukemia cells isolated from 6 AML patients with wild-type IDH2 treated ex-vivo with the indicated concentrations of DM-αKG for 6, 12 and 24 h. **g** Western blotting analysis of IDH2 and C-MYC protein expression in human primary AML cells from two patients with mutant IDH2 treated ex-vivo with indicated concentrations of DM-αKG for 6, 12 and 24 h

To further investigate how IDH2 knockdown induced downregulation of c-Myc, we tested whether the elevated α-KG could function as a metabolite to mediate c-Myc downregulation. Several human AML cell lines (ML-1, U937 and Kasumi-1) were treated with DM-αKG for various time points, and C-MYC protein expression was measured by western blotting. As shown in Fig. 7a, b, a substantial decrease in C-MYC protein was detected as early as 6 h after the leukemia cells were exposed to DM-αKG, which did not affect IDH2 protein level. The major reduction of C-MYC protein was consistently observed in all three cell lines treated with DM-αKG. Analysis of *C-MYC* mRNA showed a significant downregulation of *C-MYC* RNA in DM-αKG-treated cells (Fig. 7c). RNA stability assay showed that treatment of U937 or ML-1 cells with 4 mM DM-αKG did not cause any significant change in *C-MYC* mRNA stability (Fig. 7d), although incubation of this concentration of DM-αKG led to a major increase in α-KG in U937 cells (44 folds) and ML-1 cells (34 folds). These data together suggest that α-KG regulated c-Myc at the transcriptional level, consistent with the observations in IDH2 knockdown cells. Interestingly, when AML cells were cultured in the absence of glutamine, which caused a decrease of cellular α-KG (Fig. 3i, j), led to a substantial increase in C-MYC expression (Fig. 7e). Of note, the decrease in c-Myc in IDH2 knockdown cells could not be restored by citrate supplement (Additional file 3: Fig. S7), suggesting that the c-Myc decrease in IDH2 knockdown cells was unlikely due to the decrease of citrate levels. These data together suggest that α-KG could function as a negative regulator of c-Myc expression.

To evaluate the clinical relevance of the above findings, we tested the ability of cell-permeable α-KG to affect C-MYC expression in primary leukemia cells isolated from AML patients with known IDH2 genotypes (wild-type or mutant). We found that treatment of primary AML cells with DM-αKG led to a concentration-dependent decrease of C-MYC protein in all six patient samples with wt-IDH2 (Fig. 7f). Interestingly, in human primary AML cells with mutant IDH2, the same concentrations of DM-αKG (4–8 mM) did not cause any significant change in C-MYC protein (Fig. 7g), indicating a different metabolism in AML cells with mutated IDH2, which could produce 2-hydroxyglutarate to antagonize the function of α-KG.

### Pharmacological inhibition of wild-type IDH2 impairs AML cell viability in vitro and suppresses leukemia growth in vivo

**Fig. 8:**
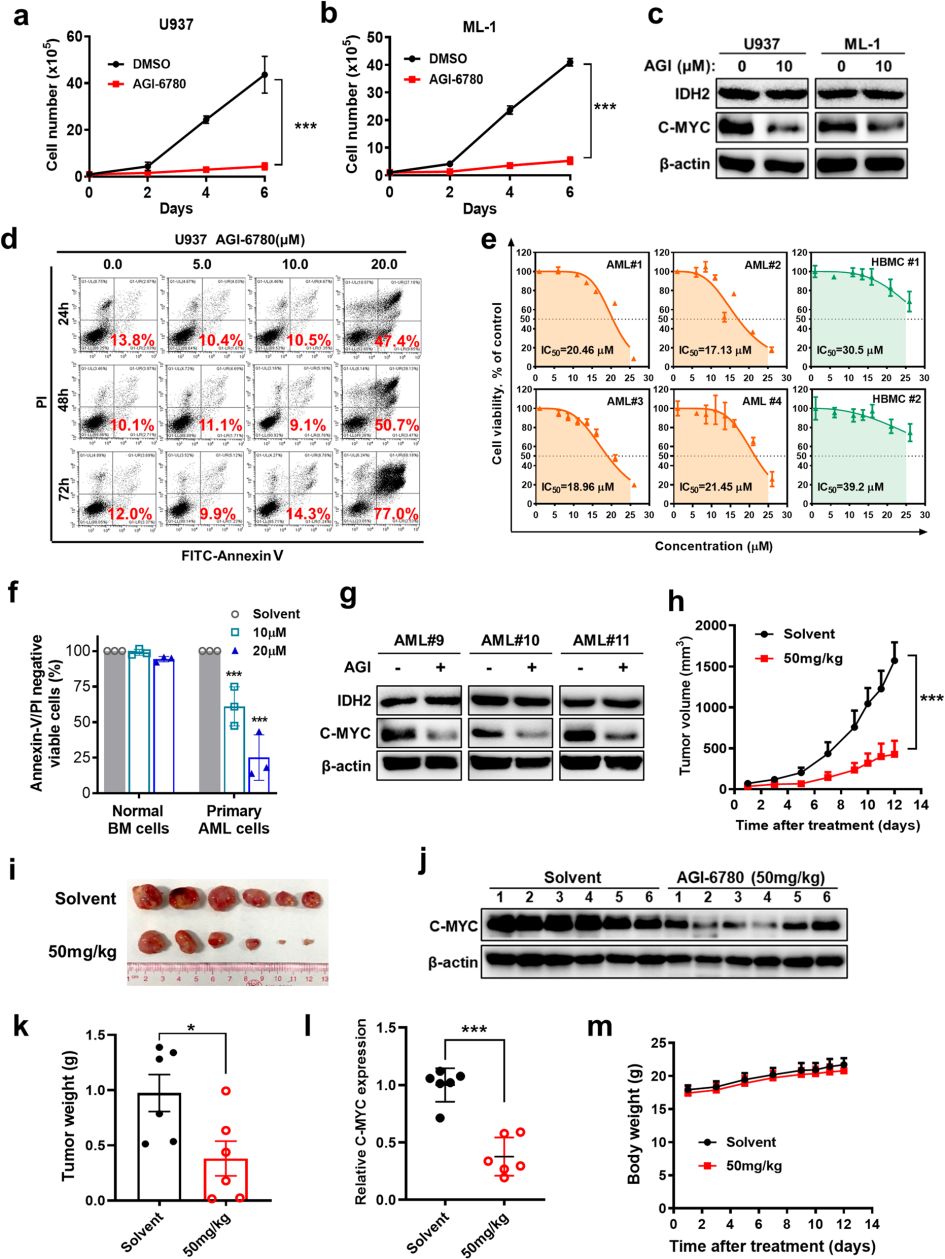
Inhibition of IDH2 by AGI-6780 suppressed AML survival in vitro and in vivo. **a**, **b** Effect of AGI-6780 on cell proliferation in U937 and ML-1 cells. Cells were treated with AGI-6780 (10 µM) or solvent (DMSO), and cell numbers were counted at the indicated time points. Data are *mean* ± *SD* of three experiments. **c** Levels of protein expression of IDH2 and C-MYC in U937 and ML-1 cells treated with AGI-6780 (10 µM) or solvent (DMSO) for 24 h. **d** The indicated cell line were treated with AGI-6780 for 72 h, and cell viability was measured using MTS assay. **e** Primary leukemia cells from AML patients with wt-IDH2 (*n* = 4) and normal human bone marrow cells (HBMC) were treated ex vivo with the indicated concentrations of AGI-6780 for 72 h, and cell viability was measured using MTS assay. **f** Comparison of percentage of Annexin V/PI-negative cells in primary AML cells and primary normal bone marrow cells treated with 10–20 µM AGI-6780 for 48 h. The original flow cytometry analysis data are presented as Additional file 3: Fig. S8c, d. **g** Protein levels of IDH2 and C-MYC in primary AML cells treated with AGI-6780 (20 µM) or with solvent (DMSO) for 24 h. **h** Growth curves of AML xenografts in athymic nude mice inoculated with ML-1 cells harboring wt-IDH2 (*n* = 6 per group, *mean* ± *SEM*). Mice were treated with daily i.p. injection of AGI-6780 or solvent as indicated. **i** Photographs of gross tumors isolated from mice at the end of the experiment. **j** Western blot analysis of C-MYC expression in tumor tissues of ML-1 xenografts isolated from mice treated with AGI-6780 or solvent as indicated. **k** Tumor weights of each group at the end of the experiment (*mean* ± *SEM*). **l** Relative C-MYC protein levels in tumor tissues of mice treated with solvent (*n* = 6) or AGI-6780 (*n* = 6). **m** Mouse body weights (*mean* ± *SD*). Two-tailed unpaired student’s *t* test for **a**, **b**, **f**, **h**, **k** and **l**, *p < 0.05, ***p < 0.001

To determine whether wt-IDH2 could be a potential therapeutic target in AML cells, we next used IDH2 inhibitor AGI-6780, a chemical compound known to potently inhibit both mutant and wild-type IDH2 [36], to treat U937 and ML-1 cell lines with wt-IDH2 and evaluated its impact on cell viability and proliferation. As illustrated in Fig. 8a, b, direct cell counting showed that inhibition of IDH2 by AGI-6780 caused a profound suppression of cell proliferation in both U937 and ML-1 cell lines. Western blotting analysis revealed a substantial decrease in C-MYC protein in AGI-6780-treated U937 and ML-1 cells (Fig. 8c), consistent with the notion that inhibition of IDH2 caused α-KG accumulation leading to downregulation of C-MYC expression. MTS assay showed that AGI-6780 induced a concentration-dependent loss of cell viability in all six leukemia cell lines tested (Additional file 3: Fig. S8a). Flow cytometry analysis showed that AGI-6780 could induce massive cell death within 24 h at a high concentration (Fig. 8d; Additional file 3: Fig. S8b). Importantly, inhibition of IDH2 by AGI-6780 exhibited potent cytotoxic effect on primary leukemia cells isolated from AML patients with wt-IDH2, while the same concentrations of AGI-6780 displayed minimum toxicity in normal human bone marrow cells using MTS assay (Fig. 8e) and flow cytometry analysis (Fig. 8f; Additional file 3: Fig. S8c, d). Consistently, we also observed a dramatic decrease in C-MYC expression in primary AML cells treated with AGI-6780 (Fig. 8g). These data suggest that wt-IDH2 could be a potential therapeutic target in AML with low toxicity to normal cells.

To further evaluate the possibility of using pharmacological inhibition of wt-IDH2 as a new treatment for AML in vivo, we tested the therapeutic efficacy of compound AGI-6780 in mice bearing subcutaneous xenografts of AML (ML-1, wt-IDH2). As shown in Fig. 8h, i, k, treatment of the mice with AGI-6780 resulted in a significant inhibition of AML xenograft growth. Western blotting analysis of protein extracts from the tumor tissues revealed that IDH2 inhibition led to a significant downregulation of C-MYC expression in vivo (Fig. 8j, l), consistent with the observation in vitro, where AML cells with IDH2 knockdown or treated with AGI-6780 exhibited a substantial reduction in C-MYC expression (Figs. 6a, b and 8c, g). There was no obvious toxic side-effect observed in the drug-treated mice, which showed no loss of body weights (Fig. 8m). These in vivo data together suggest that pharmacological inhibition of wt-IDH2 could have potential application in AML treatment, and IDH2 inhibitors such as AGI-6780 seem well tolerated in vivo.

**Fig. 9:**
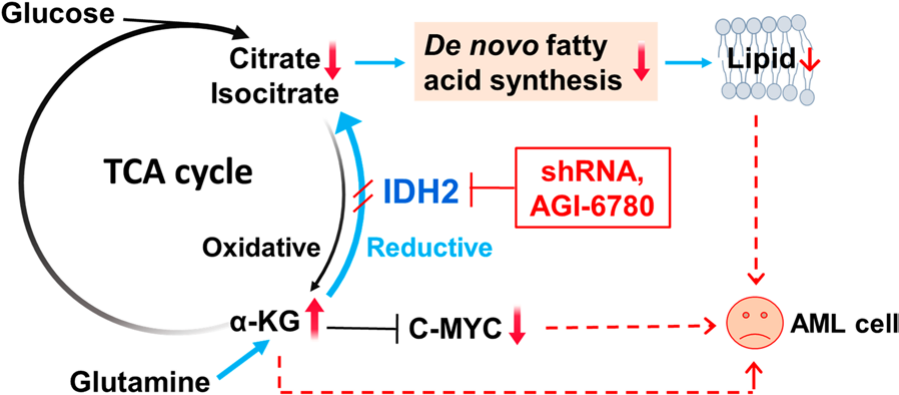
Schematic model depicting the key functions of wild-type IDH2 in AML cells and the impact of IDH2 suppression on α-KG metabolism, fatty acid synthesis, and c-Myc expression (see text for detail description)

Overall, we found that AML cells had an active reductive TCA cycle catalyzed by the highly expressed wt-IDH2, which promoted lipid synthesis and c-Myc expression through timely conversion of α-KG from glutamine to isocitrate/citrate. This biochemical property might present a metabolic vulnerability for targeting AML cells using IDH2 inhibitors (Fig. 9).

## Discussion

IDH2 is an isoform of isocitrate dehydrogenases mainly localizes in the mitochondria and catalyzes the metabolic conversion between isocitrate and α-KG in the TCA cycle, using NADP^+^/NADPH as electron acceptor/donor for the redox reaction [6, 37]. Under physiological conditions, the production of α-KG from isocitrate is an oxidative reaction, whereas the reverse metabolic flow from α-KG to isocitrate is a reductive process with a simultaneous conversion of NADPH to NADP^+^. As such, IDH2 plays a major role in both mitochondrial metabolism and redox regulation. Recent study showed that certain IDH2 mutations could change its enzymatic property, resulting in a generation of 2-HG instead of α-KG [6, 10]. Since 2-HG competes with α-KG to affect DNA and histone demethylases, the abnormal generation of 2-HG from mutant IDH2 could cause significant change in cellular epigenetics and thus induce malignant transformation. Indeed, IDH2 mutations such as IDH2-R140Q and IDH2-R172K are observed in certain subset of AML patients, and such genetic changes could co-occur with FLT3-ITD mutations in AML [38]. IDH2 inhibitors such as enasidenib (IDHIFA^®^) exhibited promising therapeutic activity in clinical trials for treatment of refractory AML patients with IDH2 mutations [39], further indicating the important role of IDH2 mutation in AML. However, the role of wild-type IDH2 in AML remains largely unknown.

Our study showed that wt-IDH2 played a major role in promoting AML cell survival and proliferation and revealed special metabolic characteristics of AML cells with respect to α-KG to isocitrate flow in the TCA cycle. The important role of wt-IDH2 in AML and its clinical relevance are supported by several lines of evidence from the current study: (1) The expression of IDH2 was elevated in AML cells and the high expression was associated with a tendency of shorter patient survival; (2) knockdown of wt-IDH2 significantly suppressed AML cell proliferation in vitro and in vivo, whereas overexpression of wt-IDH2 promoted AML cell proliferation and cell viability; (3) wt-IDH2 promoted the conversion of α-KG to isocitrate and citrate to support lipid synthesis for leukemia cell proliferation; (4) inhibition of IDH2 resulted in an abnormal accumulation of α-KG, which at high concentrations was sufficient to cause massive apoptosis in AML cells; (5) wt-IDH2 could enhance c-Myc expression to promote AML cells survival and proliferation by metabolically reducing α-KG, a metabolite capable of suppressing c-Myc expression.

One novel finding from this study was the revealing of a highly active metabolic flow from α-KG to isocitrate through reductive TCA cycle catalyzed by wt-IDH2 in AML cells. The observation that suppression of IDH2 by shRNA silencing led to a high accumulation of α-KG in AML cells was somewhat surprising, since one would predict a decrease of α-KG in the IDH2-suppressed cells based on the “normal” TCA metabolic flow from isocitrate to α-KG. The finding that inhibition of IDH2 caused an elevation of α-KG in AML cells suggests that the dominant metabolic flow catalyzed by IDH2 in AML cells was from α-KG to isocitrate in a “reverse” direction in contrast to the commonly presumed direction from isocitrate to α-KG. Interestingly, our recent study showed that IDH2 was overexpressed in colorectal cancer cells and a knockdown of IDH2 in HCT-116 and HT-29 cells led to a decrease in α-KG [40], suggesting that IDH2 might mainly catalyze the oxidative TCA metabolism in colon cancer cells. Thus, IDH2 may have different roles in a cell type-specific manner.

The biological significance of the active reverse TCA metabolic flow catalyzed by wt-IDH2 in AML seems to be multiple folds. First, the active conversion of α-KG to isocitrate and then to citrate could provide an important metabolic precursor for lipid synthesis to fulfill the need of leukemia cell proliferation, since the citrate generated in the mitochondria could translocate to cytosol via citrate shuttle as a key source of acetyl-CoA for the synthesis of fatty acids [41–44]. The active reverse (reductive) TCA cycle would enable a generation of sufficient amount of citrate from α-KG, which was likely from the active glutamine anaplerosis into the TCA cycle through glutamate dehydrogenase [43, 45]. Indeed, our metabolic flux experiment using [U-^13^C_5_] glutamine showed that suppression of IDH2 led to an accumulation in M + 5 labeling of α-KG and a decrease in M + 5 labeling of isocitrate (Fig. 4), indicating a blockage of metabolic flow from α-KG to isocitrate originated from glutamine. Furthermore, the ^13^C-labeled fatty acids from glutamine were also decreased (Fig. 4g, h), consistent with the notion that glutamine might be actively utilized in AML cells through reductive TCA cycle catalyzed by IDH2, whose inhibition led to a reduction of newly synthesized fatty acids. The decrease in lipid biosynthesis was likely an important mechanism contributing to the inhibition of AML cell proliferation induced by IDH2 suppression. Previous studies showed that rapid lipogenesis is a hallmark of most cancers including hematological malignancies and is at least partly caused by high rates of fatty acid biosynthesis [46, 47]. As such, the active reductive TCA cycle catalyzed by the highly expressed IDH2 in AML cells seems to play a major role in de novo synthesis of fatty acids essential for the proliferation of the leukemia cells.

Another biological significance of the reverse TCA cycle catalyzed by wt-IDH2 in AML is the timely conversion of α-KG to isocitrate. This active metabolic flow could prevent the accumulation of α-KG, which was shown to be toxic to AML cells at high concentrations (Fig. 5). As such, a timely metabolic removal of α-KG by IDH2 would be critical for the leukemia cell survival. This might in part explain why IDH2 expression is elevated in AML cells and its association with poor clinical outcome. The exact mechanism by which high concentrations of α-KG exert its cytotoxic effect remains unclear at this time. A recent study by Zhang et al. showed that α-KG at high concentrations (8–15 mM) could induce gasdermin C-dependent pyroptotic cell death through ROS-mediated activation of death receptor-6 and caspase-8 in Hela cells [48]. We observed that α-KG at a similar concentration range (6–10 mM) mainly caused apoptotic cell death in AML cells (Fig. 5). It is unclear at the present time if pyroptosis could also be induced by α-KG in AML cells. Further study is needed to explore this possibility.

It is important to note that either a knockdown of IDH2 or a direct addition of cell-permeable α-KG to the culture medium of AML cells (without IDH2 knockdown) could induce a significant downregulation of c-Myc expression (Figs. 6, 7). The exact mechanism by which IDH2 affects c-Myc expression is unclear at the present time. Since IDH2 knockdown could cause α-KG accumulation in AML cells and exogenous α-KG could suppress c-Myc, it is logical to postulate that IDH2 regulates c-Myc expression through α-KG. This regulation appeared to occur at the transcriptional level, as evidenced by the ability of α-KG to downregulate the expression of *C-MYC* mRNA (Fig. 7c). Consistently, IDH2 knockdown in AML cells caused α-KG accumulation and a significant decrease in *C-MYC* mRNA expression, whereas over-expression of IDH2 enhanced *C-MYC* mRNA expression (Fig. 6c–f). The ability of α-KG to regulate c-Myc expression at transcriptional level is a novel and significant finding in our study, although the exact mechanisms remain to be further defined. One possibility would be the epigenetic regulation of *C-MYC* mRNA expression by α-KG, which is known to function as a cofactor for multiple α-KG-dependent dioxygenases involved in epigenetic modification [10, 49]. Thus, it is possible that α-KG could affect DNA methylation status of c-Myc gene/promoters and impact its transcription. It is also possible that IDH2, through its metabolic impact on α-KG, might affect the expression of another transcriptional factor that regulates the expression of c-Myc. These possibilities need to be further investigated in future study. Nevertheless, the ability of α-KG to effectively downregulate c-Myc expression could be an important mechanism contributing to the cytotoxic effect of α-KG at high concentrations, since c-Myc is critical for leukemia cell survival and proliferation [31, 50]. Indeed, we showed that knocking down of c-Myc severely compromised AML cell viability and induced substantial cell death (Additional file 3: Fig. S6). Because the high expression of IDH2 in AML cells could keep α-KG at a relatively low level and thus enhance c-Myc expression, it seems possible that the elevated c-Myc expression may contribute to the pro-AML effect of IDH2.

Interestingly, a recent study indicated that C-MYC could promote IDH2 transcription associated with Epstein-Barr virus (EBV)-related tumorigenesis [51]. This observation, together with our findings that α-KG could regulate c-Myc expression, suggests a possibility that c-Myc and wt-IDH2 could form a positive feedback loop to promote leukemia development: C-MYC enhances the expression of IDH2, which converts α-KG to isocitrate and thus maintains cellular α-KG at low level. This releases the suppression on c-Myc expression, leading to elevated C-MYC, which might in turn promote IDH2 expression. Such a positive feedback loop between wt-IDH2 and C-MYC could form a vicious cycle to promote AML development.

The important role of wt-IDH2 in AML survival and proliferation suggests that this molecule could be a potential therapeutic target. Indeed, we showed that a specific knockdown of wt-IDH2 led to a potent inhibition of leukemia cell proliferation in vitro and in vivo, indicating that specific abrogation of IDH2 could exert significant therapeutic activity against AML. Importantly, pharmacological inhibition of IDH2 using small chemical compound AGI-6780 also exhibited significant inhibitory effect in AML cell lines and primary leukemia cells isolated from AML patients with wt-IDH2 in vitro and in vivo. These data together suggest that wt-IDH2 is a “druggable” target, and that it might be feasible to use pharmacological inhibitors of IDH2 for clinical treatment of AML. A significant complication, however, is that primary AML cells harboring mutant IDH2 were insensitive to the elevation of α-KG. A possible mechanistic explanation could be that AML cells with mutant IDH2 have an altered enzyme activity to produce 2-HG [10]. The high 2-HG could effectively compete with α-KG and abrogate its impact on AML cells with mutant IDH2. Our observations that DM-αKG did not cause a downregulation of c-Myc nor induce apoptosis in AML cells with mutant IDH2 seemed consistent with this possibility.

## Conclusions

In summary, our study revealed a previously unrecognized metabolic property in AML cells: an active reductive TCA metabolic flow in a “reverse” direction from α-KG to isocitrate, which enables the leukemia cells to utilize glutamine for de novo lipid synthesis. We showed that mitochondrial wild-type IDH2 plays a key role in this metabolic process and thus promotes leukemia cell survival and proliferation. We have also identified α-KG as a key metabolite that regulates the expression of c-Myc at transcriptional level. Abrogation of wild-type IDH2 caused an abnormal accumulation of α-KG, a decrease in fatty acid synthesis, and a downregulation of c-Myc expression, leading to inhibition of AML proliferation in vitro and in vivo. The observation that pharmacological inhibition of IDH2 using AGI-6780 showed significant therapeutic effect in mice-bearing AML xenografts suggests a feasibility of potential clinical application. As such, wild-type IDH2 could be a promising target for treatment of AML. Our study provides the conceptual framework and rationale for further evaluation of wild-type IDH2 inhibitors as potential drugs for the treatment of AML.

## Supplementary Information

**Additional file 1: Table S1.** Characteristics of clinical samples.

**Additional file 2: Table S2.** Reagents and materials.

**Additional file 3.** Supplementary Figures.

## Data Availability

The datasets used and analyzed during the current study are available from the corresponding author on reasonable request.

## Abbreviations

IDH2: Isocitrate dehydrogenase 2
wt: Wild-type
AML: Acute myeloid leukemia
TCA cycle: Tricarboxylic acid cycle
α-KG: Alpha-ketoglutarate
2-HG: 2-Hydroxyglutarate
FLT3-ITD: Internal tandem duplications (ITD) in fms-like tyrosine kinase 3
shRNA: Short hairpin RNA
siRNA: Small interfering RNA
KD: Knockdown
PBMCs: Peripheral blood mononuclear cells
RNA-Seq: RNA sequencing
U-^13^C_5_: Uniformly ^13^C-labeled
Gln: Glutamine
Glu: Glutamate
Isocit: Isocitrate
Cit: Citrate
AcCoA: Acetyl-coenzyme A
Fas: Fatty acid synthesis
Oac: Oxaloacetate
Mal: Malate
Fum: Fumarate
Suc: Succinate
Pyr: Pyruvate
Asp: Aspartate
IHC: Immunohistochemistry
TUNEL: Terminal deoxynucleotidyl transferase dUTP nick end labeling
OCR: Oxygen consumption rate
ATP: Adenosine triphosphate
IC_50_: Drug concentration that kills 50% of cancer cells
MTS: 3-(4,5-Dimethylthiazol-2-yl)-5-(3-carboxymethoxyphenyl)-2-(4-sulfophenyl)-2*H*-tetrazolium, inner salt
DM-αKG: Dimethyl-αKG
