# Supplementary material for: Reductive TCA cycle catalyzed by wild-type IDH2 promotes acute myeloid leukemia and is a metabolic vulnerability for potential targeted therapy
Source: J Hematol Oncol. 2022 Mar 21;15:30. doi: 10.1186/s13045-022-01245-z (PMC8935709; doi:10.1186/s13045-022-01245-z)
Supplement: Supplementary file 3 — Additional file 3. Supplementary Figures. [file 13045_2022_1245_MOESM3_ESM.pdf]

# Supplementary Figure S1

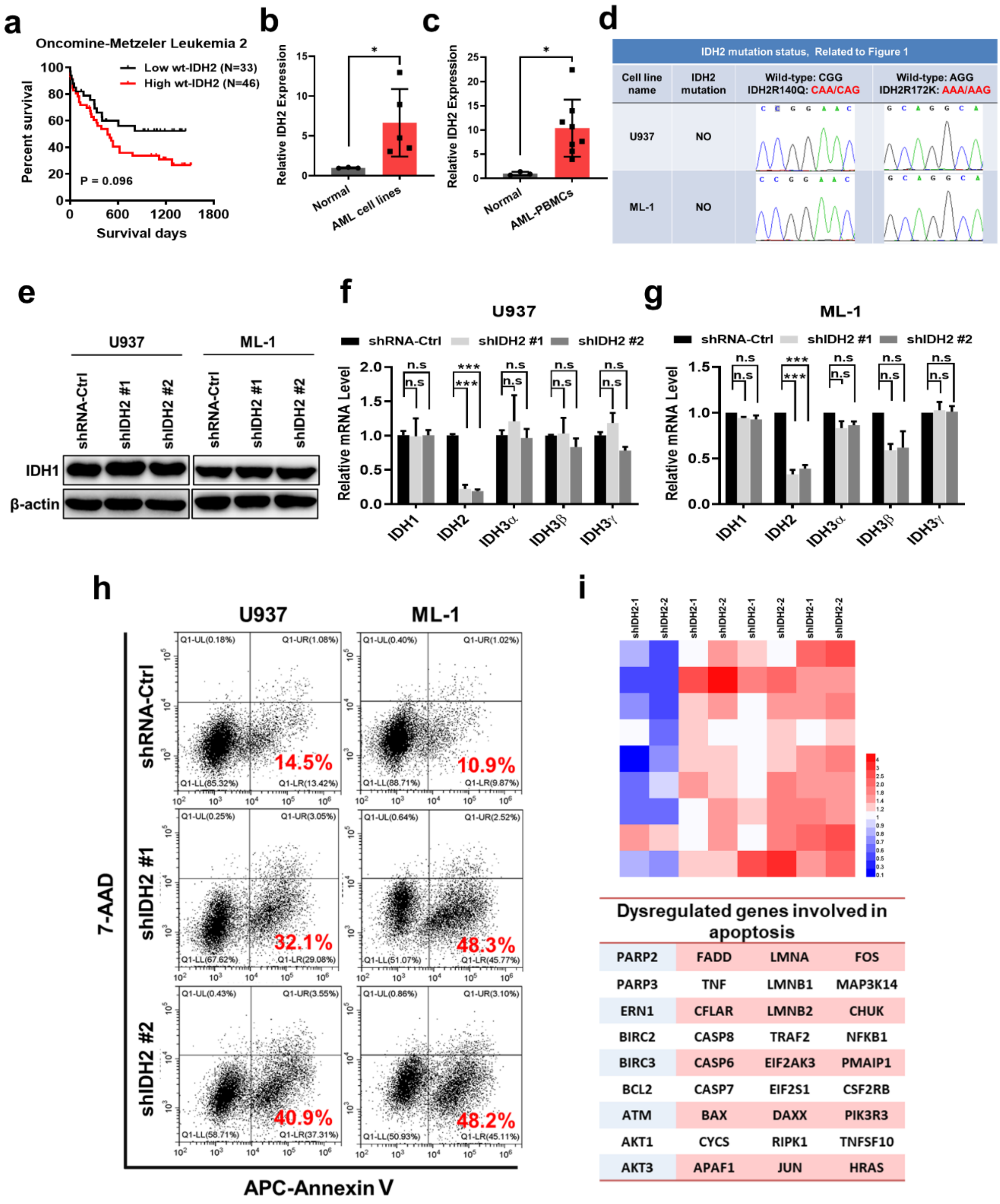


**Figure S1. Wild-type IDH2 is up-regulated and associated with worse outcomes in AML cells.**
(a) Kaplan-Meier survival curves of censored Cox analysis of AML stratified by IDH2-expression. The total number of samples analyzed was 79 for overall survival (Oncomine-Metzeler Leukemia 2). (b) IDH2 protein levels in normal human peripheral blood mononuclear cells (PBMCs, $n = 3$) and AML cell lines ($n = 5$), detected by Western blotting. (c) IDH2 protein levels in normal human PBMCs ($n = 3$) and PBMCs from AML patients with wt-IDH2 ($n = 8$), measured by Western blotting. (d) Confirmation of wt-IDH2 gene sequence in U937 and ML-1 cell lines. DNA sequences of common IDH2 mutations at amino acids 140 and 172 are also shown. (e)

Western blotting analysis of IDH1 protein levels in U937 and ML-1 cells harboring shRNA-Ctrl or shIDH2 vectors (#1 or #2). (f, g) Relative mRNA level of IDH1, IDH2, IDH3α, IDH3β and IDH3γ in U937 (f) and ML-1 (g) cells expressing shRNA-Ctrl or shIDH2 vectors (#1 or #2) ($n$ = 3). (h) Apoptosis of U937 and ML-1 cells transfected with shRNA-Ctrl or IDH2 shRNA for 48h. Apoptosis was analyzed by flow cytometry and quantified by annexin-V positivity. The number in each panel shows the percentage of dead cells. (i) Upper panel shows the heatmap of differentially expressed genes associated with apoptosis pathway. Gene expression was determined by RNA-sequencing analysis of U937 cells harboring shRNA-Ctrl or shIDH2 vectors (#1 or #2). The lower panel shows the gene names corresponding to the heatmap. *mean* ± *SD;* *$p < 0.05$, ***$p < 0.001$, *n.s*, no significance.

## Supplementary Figure S2

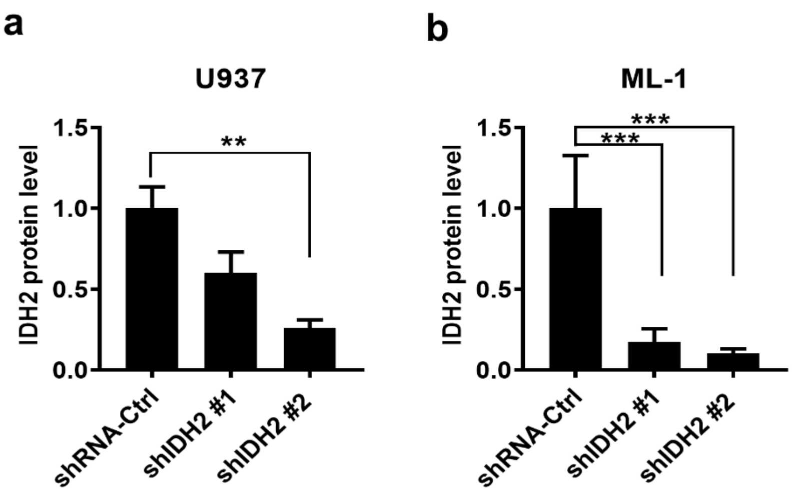


**Figure S2. Expressions of IDH2 in tumor tissues from AML xenografts with or without IDH2 stable knockdown.**

(a) IDH2 protein levels in tumor tissues from U937 xenografts harboring shRNA-Ctrl or shIDH2 vectors (#1 and #2). Protein expression was analyzed by Western blotting and quantified by densitometry analysis of the IDH2 band ($n$ = 4, *mean* ± *SD*). (b) IDH2 protein levels in tumor tissues from ML-1 xenografts harboring shRNA-Ctrl or shIDH2 vectors (#1 and #2). Protein expression was analyzed by Western blotting and quantified by densitometry analysis of the IDH2 band ($n$ = 4, *mean* ± *SD*). **$p < 0.01$; ***$p < 0.001$.

# Supplementary Figure S3

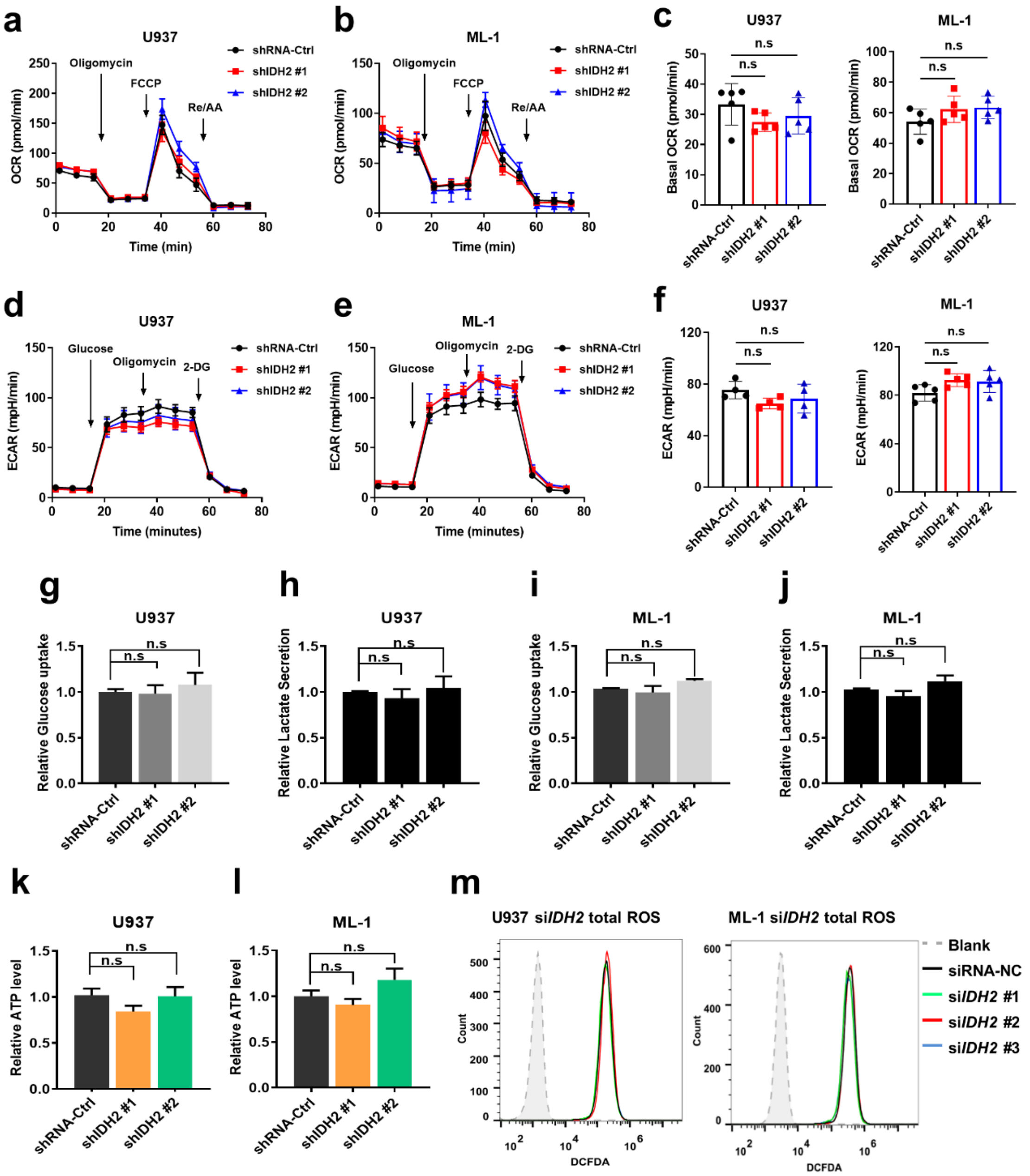


**Figure S3. Silencing of IDH2 expression did not significantly affect glucose energy metabolism.**
(a, b）Measurement of oxygen consumption rate (OCR) in U937 and ML-1 cells harboring shRNA-Ctrl or shIDH2 vectors (#1 and #2), using the Seahorse extra cellular flux analyzer. (c) Basal OCR in U937 and ML-1 cells harboring shRNA-Ctrl or shIDH2 vectors (#1 and #2). (d, e) Measurement of extra cellular acidification rate (ECAR) in U937 and ML-1 cells harboring shRNA-Ctrl or shIDH2 vectors (#1 and #2), using the Seahorse extra cellular flux analyzer. (f) Quantitation of ECAR for U937 and ML-1 cells harboring shRNA-Ctrl or shIDH2 vectors (#1 and #2). (g-i) Glucose uptake and lactate secretion by U937 (g, h) and ML-1 (i, j) cells harboring shRNA-Ctrl or shIDH2 vectors (#1 and #2). (k, l) Analysis of cellular ATP levels in U937 and ML-1 cells harboring shRNA-Ctrl or shIDH2 vectors (#1 and #2). (m) U937 and ML-1 cells were transfected with siRNA against IDH2 (siIDH2 #1-#3) or with control siRNA (siRNA-NC), cellular ROS were measured by flow cytometry using DCFH-DA staining. *mean*±*SD; n.s*, no significance.

## Supplementary Figure S4

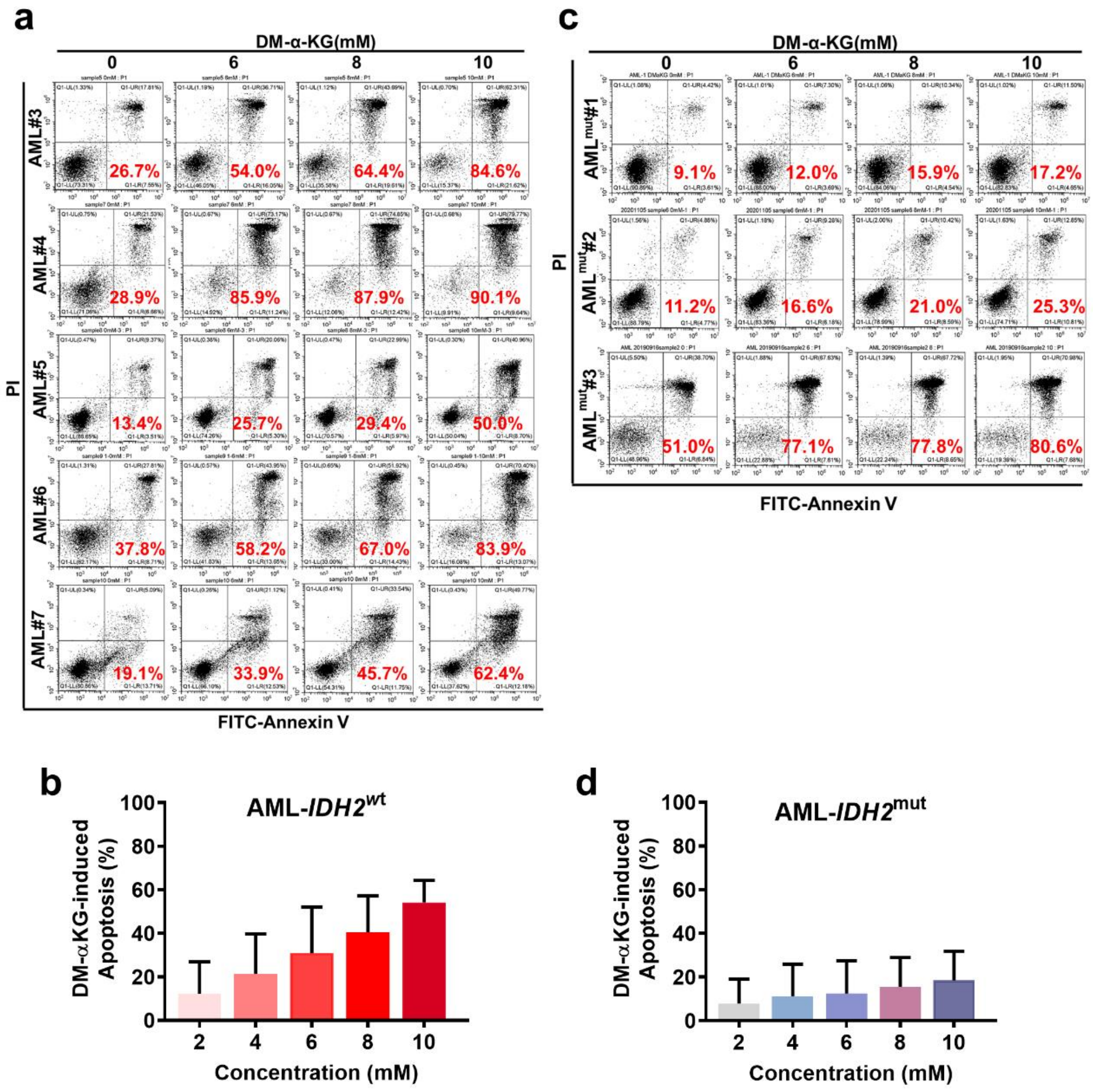


**Figure S4. Effects of α-KG on viability of AML cells with wild-type or mutant IDH2.**

(a) Primary AML cells with wt-IDH2 were treated with indicated concentrations of DM-αKG for 48 h. Apoptosis was measured by flow cytometry analysis. (b) Quantitation of apoptosis in human primary AML cells with wt-IDH2 treated with the indicated concentrations of DM-αKG ($n$ = 6, *mean* ± *SD*). (c) Primary AML cells with mutant IDH2 were treated with the indicated concentrations of DM-αKG for 48 h. Apoptosis was measured by flow cytometry analysis. (d) Quantitation of apoptosis in human primary AML cells with mutant IDH2 treated with the indicated concentrations of DM-αKG ($n$ = 3, *mean* ± *SD*).

# Supplementary Figure S5

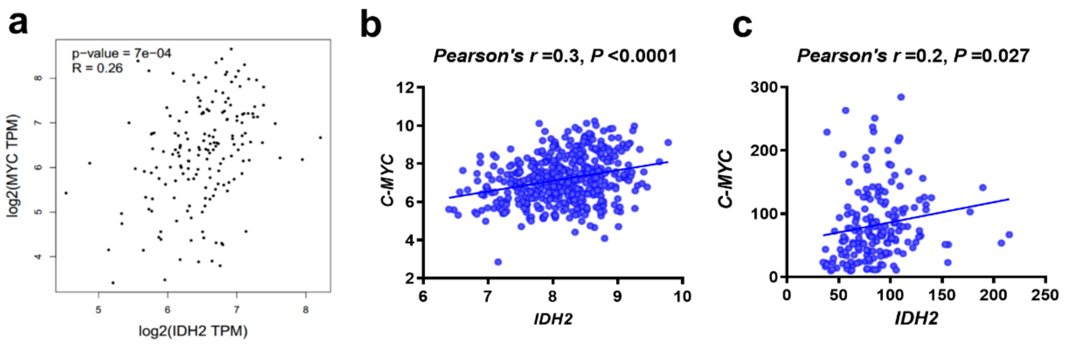


d

| U937 shIDH2 #1 | U937 shIDH2 #2 | ML-1 shIDH2 #1 | ML-1 shIDH2 #2 | Gene | HL-60 IDH2$^{OE}$ |
|---|---|---|---|---|---|
| 0.05 | 0.07 | 0.25 | 0.23 | IDH2 | 2.26 |
| 0.45 | 0.50 | 0.25 | 0.55 | C-MYC | 1.53 |
| 0.20 | 0.45 | 0.51 | 0.59 | Bcl-2 | 1.39 |
| 0.87 | 0.77 | 0.67 | 0.59 | MDM2 | |
| 0.69 | 0.41 | 0.55 | 0.33 | Cdc25a | 0.87 |
| 0.88 | 0.76 | 0.53 | 0.70 | ELK-1 | 1.06 |
| 0.34 | 0.41 | 0.54 | 0.53 | SLC7A11 | 9.33 |
| 0.50 | 0.54 | 0.66 | 0.81 | ATF4 | 3.44 |
| 0.62 | 0.52 | 0.63 | 0.42 | CCND1 | 0.68 |
| 0.74 | 0.82 | 0.76 | 0.79 | MCM7 | 1.18 |
| 0.40 | 0.59 | 0.39 | 0.60 | TFAP4 | 1.39 |
| 0.40 | 0.71 | 0.34 | 0.73 | PLD6 | 1.18 |

U937 & ML-1 with shIDH2: Relative mRNA level 0.0 0.5 1.0; *p* value 0.001 0.05

HL-60 cells with IDH2$^{OE}$: Relative mRNA level 1 5 10; *p* value 0.001 0.05

**Figure S5. Positive correlation between IDH2 and c-Myc expression.**
(a) Pearson correlation between IDH2 and C-MYC mRNA expression in AML samples from the GEPIA database (n = 173) (http://gepia.cancer-pku.cn/detail.php). (b) Pearson correlation between IDH2 and C-MYC mRNA expression in IDH2wt AML samples from cBioPortal database (n = 451) (http://www.cbioportal.org/study/summary?id=aml_ohsu_2018). (c) Pearson correlation between IDH2 and C-MYC mRNA expression in IDH2wt AML samples from cBioPortal database (n = 179) (http://www.cbioportal.org/study/summary?id=laml_tcga). (d) Heatmap of mRNA levels of C-MYC target genes determined by RT-qPCR in AML cells with IDH2 shRNA (normalized by shRNA-Ctrl cells) or in HL-60 cells with IDH2$^{OE}$ (normalized to by vector-Ctrl cells). Data are shown as mean values of 3 independent experiments.

## Supplementary Figure S6

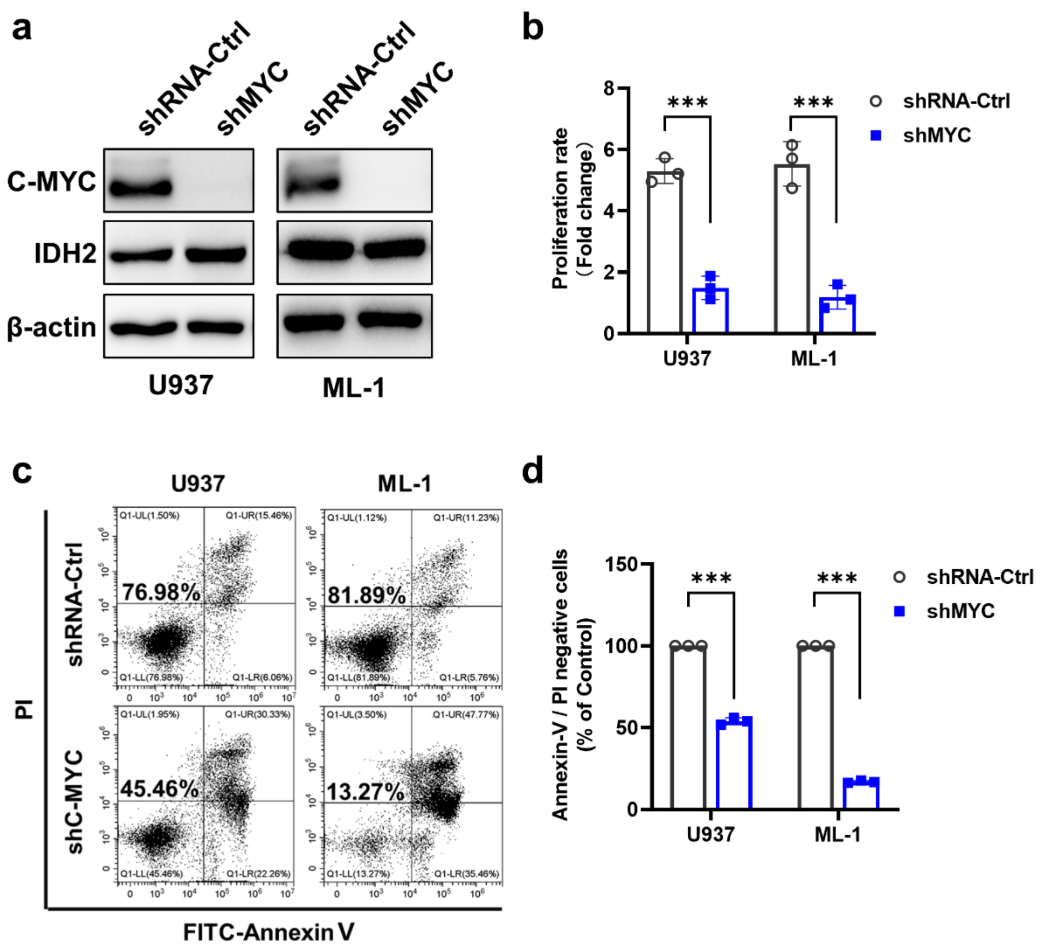


**Figure S6. Effects of c-Myc knockdown on IDH2 expression and survival of AML cells.**

(a) Western blot analysis of IDH2 protein levels in U937 and ML-1 cells transfected with shRNA-Ctrl or C-MYC shRNA. (b) Proliferation rate of U937 and ML-1 cells transfected with shRNA-Ctrl or C-MYC shRNA at day 3 divided by day 1. (c, d) Apoptosis of U937 and ML-1 cells transfected with shRNA-Ctrl or C-MYC shRNA for 48 h using flow cytometry analysis of annexin-V positivity. The number in each panel shows the percentage of AnnexinV/ PI negative cells. $n = 3$, *mean* ± *SD* , $^{***}p < 0.001$.

## Supplementary Figure S7

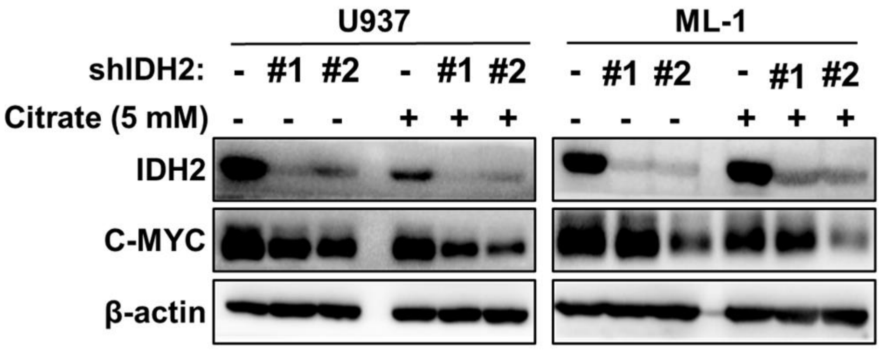


**Figure S7. Impact of citrate supplement on the expression of IDH2 and C-MYC in AML cells.**
U937 and ML-1 cells harboring shRNA-Ctrl or shIDH2 vectors (#1 and #2) were incubated with or without sodium citrate (5 mM) for 24 h. Expression of IDH2 and C-MYC was measured by Western blotting.

## Supplementary Figure S8

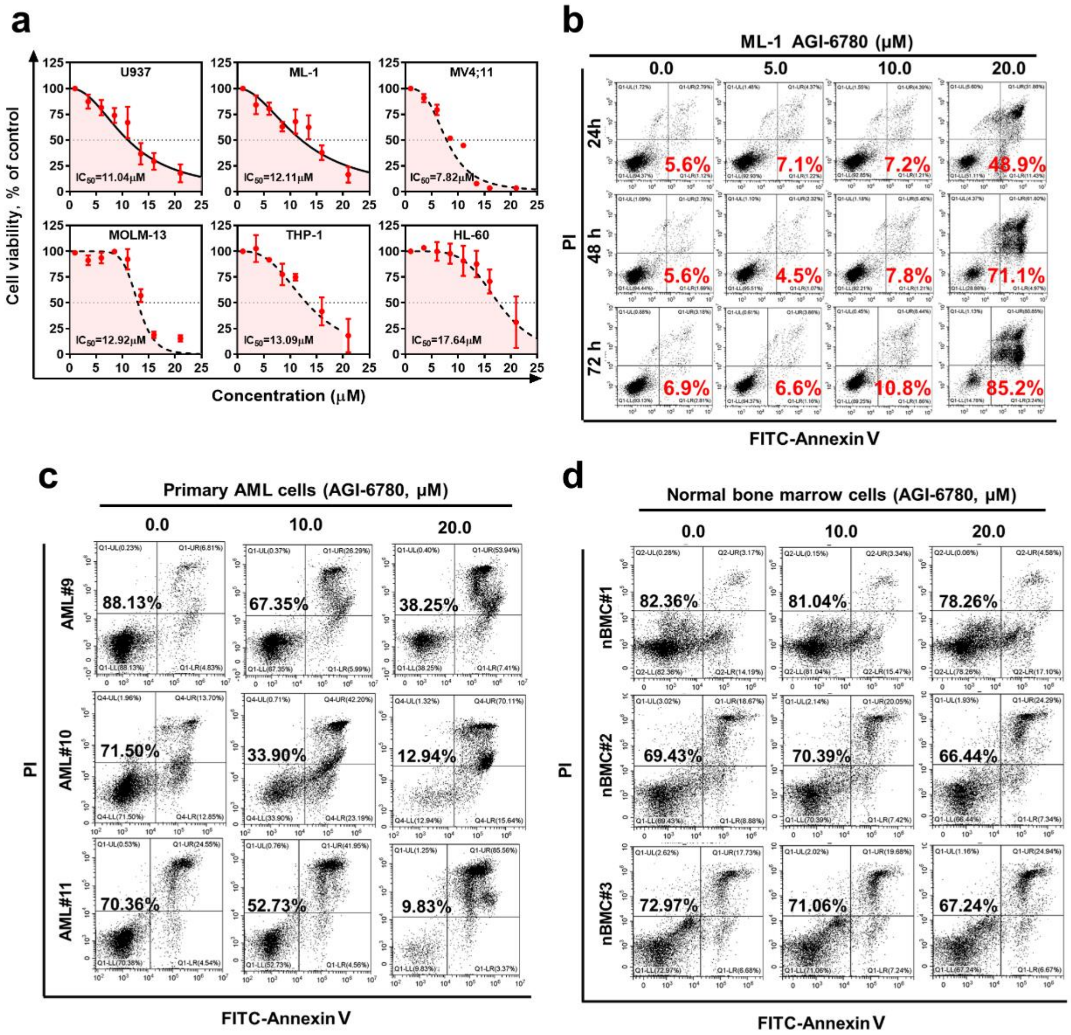


**Figure S8. Effect of IDH2 inhibitor AGI-6780 on AML cell viability.**

(a) The indicated cell lines were treated with AGI-6780 for 72 h, and cell viability was measured using MTS assay. (b) Apoptosis induced by AGI-6780 in ML-1 cells. Cells were treated with 5-20 µM AGI-6780 for 24, 48 and 72 h, apoptosis was measured using flow cytometry analysis of annexin-V positivity. The number in each panel shows the percentage of dead cells. (c, d) Apoptosis induced by AGI-6780 in primary AML cells and normal bone marrow cells. Cells were treated with 10 and 20 µM AGI-6780 for 48 h, apoptosis was measured using flow cytometry analysis of annexin-V positivity. The number in each panel shows the percentage of AnnexinV/ PI negative cells.
